# Translational regulation shapes the molecular landscape of complex disease phenotypes

**DOI:** 10.1038/ncomms8200

**Published:** 2015-05-26

**Authors:** Sebastian Schafer, Eleonora Adami, Matthias Heinig, Katharina E. Costa Rodrigues, Franziska Kreuchwig, Jan Silhavy, Sebastiaan van Heesch, Deimante Simaite, Nikolaus Rajewsky, Edwin Cuppen, Michal Pravenec, Martin Vingron, Stuart A. Cook, Norbert Hubner

**Affiliations:** 1 Cardiovascular and Metabolic Sciences, Max-Delbrück-Center for Molecular Medicine (MDC) in the Helmholtz Association, Robert-Rossle-Strasse 10, 13125 Berlin, Germany; 2 National Heart Research Institute Singapore (NHRIS), National Heart Centre Singapore, Singapore 169609, Singapore; 3 Department of Computational Molecular Biology, Max Planck Institute for Molecular Genetics, Ihnestrasse 63-73, 14195 Berlin, Germany; 4 Institute of Physiology, Academy of Sciences of the Czech Republic, Vídenska 1083, 142 20 Prague 4, Czech Republic; 5 Systems Biology of Gene Regulatory Elements, Max-Delbrück-Center for Molecular Medicine (MDC) in the Helmholtz Association, Robert-Rossle-Strasse 10, 13125 Berlin, Germany; 6 DZHK (German Centre for Cardiovascular Research), Partner Site, 13347 Berlin, Germany; 7 Hubrecht Institute-KNAW & University Medical Center Utrecht, Uppsalalaan 8, 3584 CT Utrecht, The Netherlands; 8 National Heart and Lung Institute, Imperial College London, London SW3 6NP, UK; 9 Duke-National University of Singapore, Singapore 169857, Singapore; 10 Charité-Universitätsmedizin, 10117 Berlin, Germany

## Abstract

The extent of translational control of gene expression in mammalian tissues remains largely unknown. Here we perform genome-wide RNA sequencing and ribosome profiling in heart and liver tissues to investigate strain-specific translational regulation in the spontaneously hypertensive rat (SHR/Ola). For the most part, transcriptional variation is equally apparent at the translational level and there is limited evidence of translational buffering. Remarkably, we observe hundreds of strain-specific differences in translation, almost doubling the number of differentially expressed genes. The integration of genetic, transcriptional and translational data sets reveals distinct signatures in 3′UTR variation, RNA-binding protein motifs and miRNA expression associated with translational regulation of gene expression. We show that a large number of genes associated with heart and liver traits in human genome-wide association studies are primarily translationally regulated. Capturing interindividual differences in the translated genome will lead to new insights into the genes and regulatory pathways underlying disease phenotypes.

## Abstract (web summary)

To what extent translational control can contribute to global gene expression patterns in the disease state is poorly defined. Here the authors conduct genome-wide RNA-seq and ribosome profiling in a rat model of hypertension and uncover altered translation patterns in disease associated genes.

## Graphical abstract

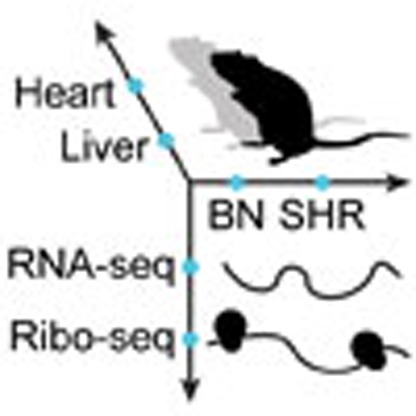

Heritable RNA expression phenotypes have been studied extensively^1^,^2^, but the contribution of translational regulation to natural phenotypic variation is largely unknown. Several genetic disorders are believed to be caused by mutations that affect protein translation^3^, but so far genome-wide translation has not been studied in complex disease models. It remains a matter of debate how well transcript abundances explain protein levels^4^,^5^, to what extent RNA expression differences are mirrored by the proteome^6^,^7^,^8^,^9^,^10^ and how many genes are under translational control in mammalian tissues.

To obtain quantitative measurements of translation, we adapted ribosome profiling procedures^11^,^12^ to reliably isolate and sequence ribosome-protected RNA fragments (RPFs) from whole-tissue samples (Ribo-seq). We then investigated translational regulation in the heart and liver of two inbred rat strains, the BN-*Lx* reference strain (*n*=5) and the SHR/Ola (*n*=5), a widely studied model of cardiovascular and metabolic disease traits^2^,^13^,^14^,^15^,^16^,^17^.

Ribosome profiling of disease tissue reveals widespread interindividual regulation of translation that shapes the molecular landscape of the cardiometabolic phenotype of the SHR/Ola rat. Ribo-seq data are a better proxy for protein levels compared with RNA-seq data and expose almost twice as many differences in gene expression between strains. The integration of transcriptional and post-transcriptional control of gene expression identifies distinct molecular signatures and reveals the contribution of genetic variation, microRNAs (miRNAs) and RNA-binding proteins (RBPs) to translational regulation. Many genes associated with cardiac and hepatic traits in humans are translationally, and not transcriptionally, regulated in disease tissue.

## Results

### RNA transcription and translation in rat heart and liver

**Figure 1:**
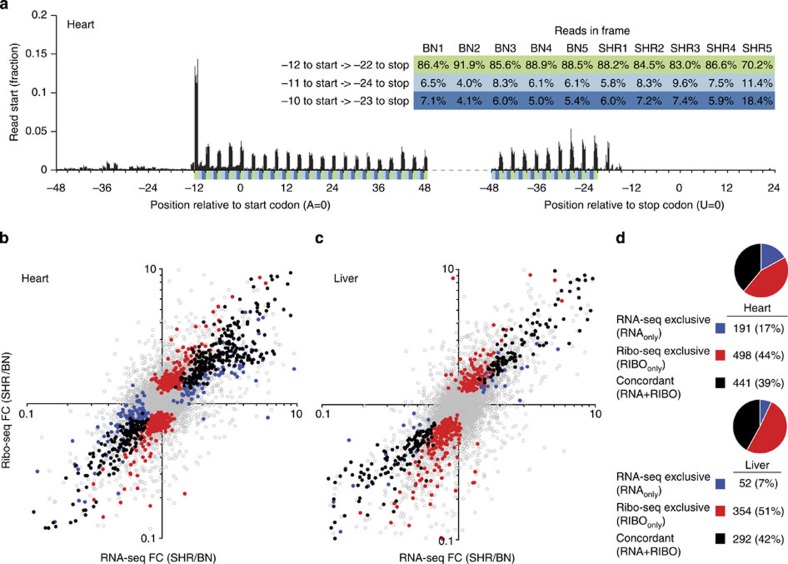
RNA-seq and Ribo-seq of cardiac and hepatic tissue. (**a**) Start positions of RNA fragments protected by ribosomes (total of 10 cardiac Ribo-seq libraries, each plotted individually). Ribosomes are located at the start codon and then move sequentially along the triplet code to translate the genetic message (for liver see Supplementary Fig. 1d). Once the ribosomal site occupied by aminoacyl-tRNA (A site) reaches the stop codon, ribosomes are released from the transcripts. For each library, the fraction of reads covering each frame is shown. The majority of ribosomes are located on the codons of the open-reading frame of protein-coding genes. (**b,c**) Differential expression between BN-*Lx* and SHR/Ola rat strains (five biological replicates each) in (**b**) heart and (**c**) liver. Fold changes of RPF and RNA abundance correlate on a genome-wide scale (Pearson's, *P*<1e−4). While the majority of genes showed no significant inter-strain differences (grey dots, *n*_heart_=9,856, *n*_liver_=9,586), hundreds of genes were differentially expressed (FDR≤0.01) at the level of transcription only (blue dots, RNA_only_), translation only (red dots, RIBO_only_) or both (black dots, RNA+RIBO). (**d**) Pie charts and tables indicate for each tissue the fractions and absolute numbers of RIBO_only_, RNA_only_ and RNA+RIBO genes of differentially expressed genes, respectively.

The Ribo-seq libraries allowed us to monitor translating ribosomes at sub-codon resolution. We observed codon periodicity (Fig. 1a) and other attributes (Supplementary Fig. 1) that are characteristic for *in vivo* snapshots of active translation. To distinguish translational from transcriptional regulation of gene expression, we integrated two distinct RNA-seq experiments: one polyA-selected data set (RNA-seq1) and one ribosomal RNA (rRNA)-depleted data set (RNA-seq2), which was generated in parallel to ribosome profiling from the identical tissue lysate. To adjust for variation in sequencing depth across technologies, mappability and fragment size, we matched the read length and the number of uniquely aligning reads located within exons for all data sets (Supplementary Fig. 2). To exclude strain-specific mapping biases, we used the SHR/Ola and BN-*Lx* genome sequence information^18^,^19^,^20^ and mapped the reads of each strain to its respective genome. We defined significant strain-specific differences in RPF and RNA expression levels (false discovery rate (FDR)≤0.01) using DESeq2 (ref. 21) (Supplementary Fig. 3).

We compared differences in messenger RNA (mRNA) levels to variation in RPF abundances between strains (Fig. 1b–d; Supplementary Fig. 4). Three classes of genes represent different modes of regulation (Supplementary Data 1 and 2): (i) Forwarded inter-strain differences in RNA expression that were carried onward to translation (RNA+RIBO; *n*_liver_=292; *n*_heart_=441); (ii) buffered RNA expression differences that were not detected on the level of translation but were present in both RNA-seq experiments (RNA_only_; *n*_liver_=52; *n*_heart_=191); (iii) reinforced strain-specific differences that were not detected in either RNA-seq experiments but were apparent at the translational level and thus exclusive to ribosome profiling data (RIBO_only_; *n*_liver_=354; *n*_heart_=498).

Whereas in yeast a variable extent of translational buffering was observed^22^,^23^,^24^, the majority of RNA expression differences were forwarded to the level of translation in mammalian heart (70%) and liver (85%). In addition, we detected hundreds of strain-specific differences in mRNA translation that were not observed at the RNA level almost doubling the number of differentially expressed genes found between strains compared with RNA-seq data alone.

**Figure 2:**
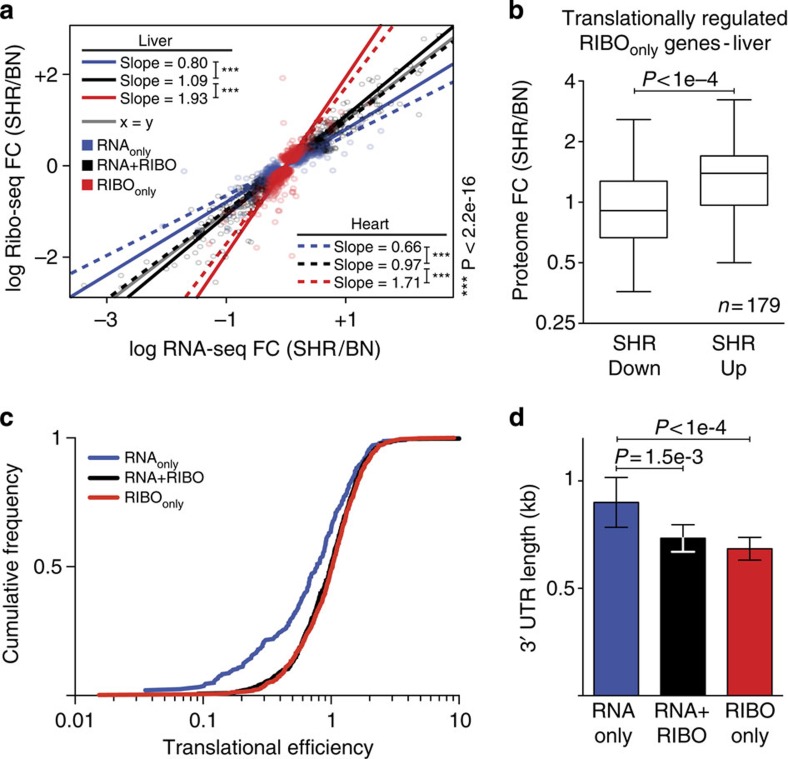
Characteristics of genes under translational regulation. (**a**) Black slopes approximated 1 (x=y) and indicated equal fold changes of expression differences between SHR/Ola and BN-*Lx* across both levels of regulation for RNA+RIBO genes (standardized major-axis estimation; SMA). Significant (*P*<2.2e−16) divergence of blue and red slopes demonstrated translational regulation for RNA_only_ and RIBO_only_ genes, respectively. (**b**) Strain-specific differences of translationally regulated RIBO_only_ (*n*=179) genes were confirmed in the liver proteome (Wilcoxon-Mann-Whitney; *P*<1e−4). Variation in translation captured by Ribo-seq resulted in significantly different protein abundances across strains (whiskers indicate 5th and 95th percentiles). RNA_only_ genes have a lower (**c**) translational efficiency and a significantly increased (**d**) 3′UTR length compared with RIBO_only_ and RNA+RIBO genes (mean with 95% confidence interval).

We then quantified the amount of translational regulation by calculating the slopes (standardized major-axis estimation)^25^ between RNA-seq and Ribo-seq fold changes between strains for genes with differential expression (Fig. 2a). This enabled us to determine the global contribution of translational regulation to strain-specific differences in gene usage in addition to transcriptional regulation. For RNA+RIBO events, transcriptional and translational differences co-occurred and slopes approximated 1 (slope_heart_=1.09; slope_liver_=0.97). Overall, the majority of RNA expression differences in tissues are associated with equal variation in RPF abundance of the same magnitude and direction.

The slopes for the RIBO_only_ genes were significantly greater than RNA+RIBO slopes (likelihood ratio test; slope_heart_=1.71; slope_liver_=1.93; *P*<2.2e−16) for both tissues, indicating reinforced strain differences that arise through post-transcriptional control.

### Translational regulation shapes the proteome

To assess whether genome-wide strain-specific differences in RPF abundance were indeed reflected on the proteome, we integrated two mass-spectrometry based quantitative proteome data sets from liver of SHR/Ola and BN-*Lx* rat strains^26^.

We globally assessed whether our data supported the expected flow of genetic information from transcription through translation to protein levels. Each step of biological information processing introduces a new set of specific regulatory changes (for example, post-transcriptional or -translational) that affect the abundance of a gene product. However, consequences of events that occur in later stages are not expected to be evident in preceding stages. In particular, translation levels reflect both transcriptional and translational regulation events, whereas transcript levels only reflect transcriptional—and not translational—regulation events. Therefore, when comparing RNA levels and protein levels conditioning on the translation levels, we would expect no additional information about the protein levels to be present in the RNA and thus they should be conditionally independent. On the other hand, when comparing translation levels and protein levels conditioning on the RNA levels, we would still expect dependences between translation levels and protein levels due to translational regulation events. We first tested the conditional independence between RNA and protein levels, given the Ribo-seq data using partial correlations^27^. In both strains the partial correlation was not significantly different from zero (Supplementary Table 1). Second, for each strain we found highly significant partial correlations between Ribo-seq data and protein levels when we conditioned on RNA transcript levels (Supplementary Table 1). This corroborated previous observations^11^ that ribosome footprint abundances correlated better with genome-wide protein levels than RNA-seq data, and indicated that Ribo-seq provides a better proxy for protein levels than RNA-seq in our data (Supplementary Fig. 5). Transcript levels provided no additional information to explain protein expression. Strain-specific regulation of translation (RIBO_only_), not detected by RNA expression profiling, was found to account for significant differences in protein abundances across strains (Fig. 2b; Wilcoxon–Mann–Whitney; *P*<1e−4). Ribo-seq data effectively revealed additional variation in gene usage that explains protein levels (Supplementary Fig. 6).

For 25% of differentially transcribed genes across both tissues, we did not detect strain-specific differences in RPF abundances. This can in part be explained by translational buffering of RNA expression differences for these RNA_only_ genes (likelihood ratio test; slope_heart_=0.66; slope_liver_=0.8; *P*<2.2e−16), reducing the inter-strain variation in gene expression. In addition, RNA_only_ genes were translated at a low efficiency (Fig. 2c), resulting in fewer ribosome footprints per transcript. Translational efficiency (TE) is defined as the number of RPFs compared with RNA-seq reads covering the coding sequence (CDS) of genes (see Methods). Low TE effectively reduced the power to detect differences using Ribo-seq compared with RNA-seq. Thus, these genes cannot be as effectively quantified during and after translation, which may result in an overestimation of buffering events. RNA_only_ genes possess significantly longer 3′untranslated regions (UTRs) (Fig. 2d; Wilcoxon–Mann–Whitney test; *P*_red_<1e−4; *P*_black_=1.5e−3), which has been implicated previously with low TE^28^,^29^.

### Distinct regulation across levels of gene expression

**Figure 3:**
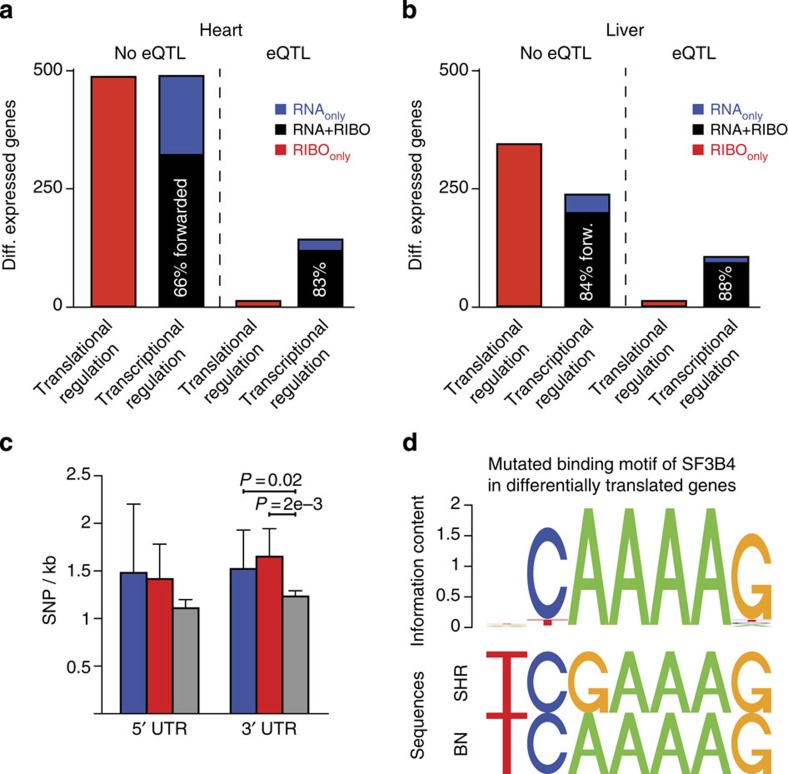
Genetic variation and strain-specific differences in translation. (**a**,**b**) Linkage analysis using RNA-seq data of the HxB/BxH recombinant inbred panel^31^ derived from the BN-*Lx* and SHR/Ola strains revealed RNA expression differences under genetic control (eQTLs) for the (**a**) heart and the (**b**) liver. Genes with differential RNA expression (forwarded: black bar; buffered: blue bar) between the parental strains were enriched for eQTLs. As expected, genes regulated on the translational level only (red bar) were not significantly enriched for eQTLs (for details see Supplementary Table 2). The majority of RNA expression differences were forwarded to the translational level and detected in both RNA-seq and Ribo-seq experiments. This percentage increased when RNA expression differences were under genetic control. (**c**) SNP density in the regulatory regions of RNA_only_ (blue bar) and RIBO_only_ (red bar) genes compared with genes without RNA or RPF strain-specific differences (grey bar). SNP density was significantly higher in the 3′UTR of translationally regulated genes (Wilcoxon–Mann–Whitney). Error bars indicate 95% confidence interval. (**d**) Known motifs of RNA-binding proteins such as *Sf3b4* were more often altered by sequence variants in the 3′UTR compared with genes that did not undergo translational regulation.

It is a matter of debate how well inherited RNA expression traits (eQTLs) are mirrored by the proteome and how much post-transcriptional processes contribute. Previous studies suggested that ∼32–35% of eQTLs lead to inherited differences in protein abundance^10^,^30^ or more recently, green fluorescent protein-tagged^7^,^8^ or micro-western array-based approaches^9^ focused on subsets of proteins and show that more than 50% of eQTLs also have a corresponding protein QTL. Here, we assessed the proportion of eQTLs that are forwarded to differential translation at the genome-wide level in mammalian tissues. We previously performed RNA expression linkage analysis of the HxB/BxH recombinant inbred panel derived from the parental BN-*Lx* and SHR/Ola strains^31^ (see Methods). Of 489 differentially transcribed genes in the heart, 143 have an eQTL (*χ*^2^-test; *P*_heart_<2.2e−16; in liver: 106 out of 238; *P*_liver_<2.2e−16; see also Supplementary Table 2). More than 80% of genes with an eQTL are differentially translated (Fig. 3a) between the two parental strains, demonstrating that genetically induced RNA expression changes are largely carried forward to differences in RPF levels and are not frequently buffered during translation. We also detected hundreds of RIBO_only_ events that do not overlap with eQTLs and yet affect protein abundance in the parental strains (Fig. 3a,b). These post-transcriptional gene expression differences cannot be captured by traditional eQTL approaches but may likely contribute to phenotypic variation.

Accurate quantification of translation in the context of natural phenotypic variation created the opportunity to begin to identify regulators of post-transcriptional processes. We observed an increase in the single-nucleotide polymorphism (SNP) density in the 3′UTR of genes under translational control (Fig. 3c), suggesting the presence of genetic variation in *cis*-regulatory elements. We tested whether known motifs of RBPs are more often altered by sequence variants in the 3′UTR of RIBO_only_ genes compared with genes that did not undergo translational regulation (RNA+RIBO). The binding sites of known translational regulators such as CPEB3 (ref. 32) (Wilcoxon–Mann–Whitney test; *P*_corrected_=0.026) were significantly enriched for genetic variation, but the analysis also suggested a role for splicing factors such as SF3B4 (Wilcoxon–Mann–Whitney test; *P*_corrected_=0.033) as well as other factors in translation by binding to *cis*-regulatory elements (Fig. 3d; see also Supplementary Tables 3 and 4).

miRNAs are known regulators of gene expression by binding preferentially to the 3′UTR, and while some miRNAs mainly act by decreasing RNA levels to reduce protein expression^33^, others act first to regulate translation^34^. To globally assess the contribution of natural variation in miRNA levels to transcriptional and post-transcriptional regulation in the heart and liver, we performed genome-wide sequencing of miRNAs in both tissues from SHR/Ola (*n*=4) and BN-*Lx* rat strains (*n*=4). Binding sites for differentially transcribed miRNAs (FDR<0.05) were enriched in the 3′UTRs of differentially translated (RIBO_only_) genes when compared with differentially transcribed (RNA+RIBO) genes (*χ*^2^-test; *P*=0.008; Supplementary Table 5). This may suggest a widespread role of miRNAs in strain-specific translational regulation in mammalian tissues.

## Discussion

We aimed to estimate the relative contribution of translational and transcriptional regulation to the expression differences in our rat model for cardiac and metabolic disease. First, we performed gene set enrichment analyses to identify disease-specific pathways that are differentially regulated between strains (Supplementary Data 3–5). Of 37 and 31 enriched KEGG (kyoto encyclopedia of genes and genomes) pathways in the heart and liver, respectively, the majority, 70.3% in the heart and 77.4% in the liver, were only detected with Ribo-seq data. Thus, the investigation of Ribo-seq data revealed a large proportion of potentially disease-specific variation that was undetected by mRNA expression analysis. Important dysregulated pathways that were only found by ribosome profiling included ‘fatty acid metabolism' in the heart and liver (hypergeometric test; *P*_heart_=1e−4; *P*_liver_=5.2e−6) and ‘hypertrophic cardiomyopathy' in the heart (hypergeometric test; *P*_heart_=1.6e−5) (Supplementary Fig. 7). These findings may warrant further investigation and suggest that the penetrance of genetic risk variants may be affected by protein variation.

**Figure 4:**
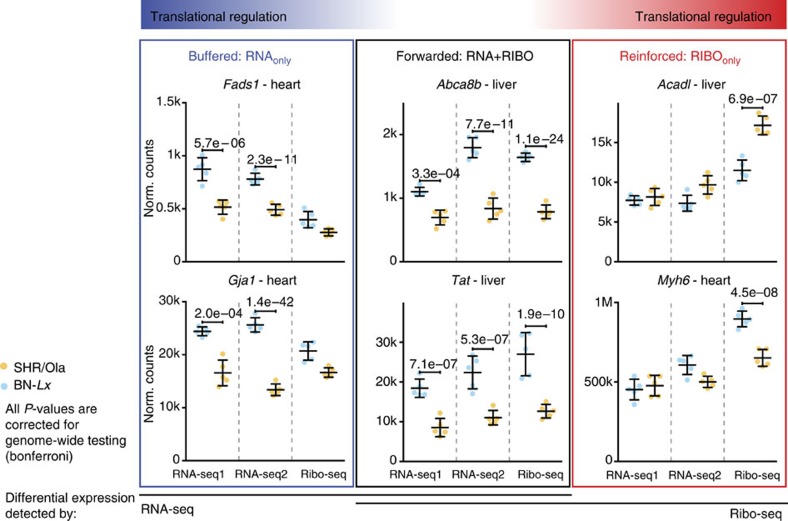
Genes associated with human cardiac and hepatic complex traits are under transcriptional and/or translational control. The SHR/Ola rat strain is an animal model for traits investigated in human GWAS studies. Genes associated with ‘heart rate': *Myh6*, *Gja1* and *Fads1* (ref. 36), ‘metabolite levels': *Acadl*, *Tat*^37^,^38^,^60^,^61^,^62^ or ‘high-density lipoprotein cholesterol': *Abca8b*^63^,^64^,^65^ are under transcriptional and/or translational control. Representative examples of two genes for each group (RNA_only_, RNA+RIBO and RIBO_only_) are shown. For each gene, read counts for five biological replicates per strain across three sequencing experiments are plotted: ribosome profiling (Ribo-seq), polyA-selected RNA sequencing (RNA-seq1) and rRNA-depleted total RNA sequencing (RNA-seq2). Error bars indicate s.d. *P* values were corrected for multiple testing and indicate significant differences in expression (DESeq2 (ref. 45); FDR≤0.01).

Genome-wide association studies (GWASs) revealed numerous genes associated with common complex diseases^35^. We analysed candidate genes from previously identified human GWASs to investigate their mode of regulation in disease-relevant tissues in the rat. Despite known pathophysiological differences between rats and humans, the SHR represents a widely used model for common complex cardiac and hepatic phenotypes. Figure 4 provides several examples where we documented strain-specific transcriptional and/or translational regulation in the liver or heart of human GWAS candidate genes that were identified for cholesterol, metabolite and heart rate phenotypes. For example, gene expression of *Myh6* (GWAS candidate gene for heart rate)^36^, and *Acadl* (GWAS candidate gene for metabolite levels and implicated in hepatic insulin resistance)^37^,^38^, is regulated at the translational level only between rat strains (Fig. 4). GWASs have identified numerous SNPs that are associated with common complex traits and diseases but their functional role is often elusive although regulation of RNA expression levels has most often been implicated to explain genotype–phenotype associations^39^. Given the extent of translational regulation of GWAS candidates (Supplementary Table 6) identified in this study, we anticipate that many GWAS variants will influence translation. Mining this additional layer of regulatory information will likely lead to new mechanistic insights into human disease susceptibility and severity.

## Methods

### Animal tissues

Heart and liver tissues for the experiments described in this study were harvested from 6-week-old unfasted BN-*Lx* and SHR/Ola male rats between 0900 and 1000 hours and immediately snap frozen in liquid nitrogen^2^. We processed 5 biological replicates for each strain and tissue type to accurately assess biological variation in expression levels. Animals were housed, bred and fed *ad libitum* in an air-conditioned animal facility at the Czech Academy of Sciences, Prague, Czech Republic. All experimental procedures were carried out in accordance with the European Union National Guidelines and the Animal Protection Law of the Czech Republic (311/1997) and were approved by the Ethics Committee of the Institute of Physiology, Czech Academy of Sciences, Prague.

### Parallel generation of ribosome profiling and RNA libraries

For each animal ∼100 mg of frozen tissue were pulverized manually under liquid nitrogen and lysed in 1 ml lysis buffer (1 × ARTseq mammalian polysome buffer (Epicentre), 1% Triton X-100, 0.1% NP-40, 1 mM dithiothreitol, 10 U ml^−1^ DNase I and nuclease-free H_2_O to final volume). To impede post-lysis translation, the lysis buffer was supplemented with cycloheximide (Sigma), previously dissolved in EtOH, at a final concentration of 0.1 mg ml^−1^. The tissue was homogenized further by repeatedly passing the lysate through a 21-gauge syringe needle. For complete lysis, the samples were kept on ice for 10 min and subsequently centrifuged at 20,000*g* to precipitate cell debris.

To accurately dissect translation and transcription, we prepared both Ribo-seq and RNA-seq libraries for each biological replicate from the identical lysate. Ribosome footprints were generated by treating part of the lysate with proprietary ARTseq nuclease (Epicentre). We then purified monosomes through Sephacryl S400 columns (GE Healthcare) and extracted RPFs with phenol chloroform. Ribosomal RNA was removed using the RiboZero Gold Magnetic Kit (Epicentre) before polyacrylamide gel electrophoresis (PAGE) purification. Sequencing adapters were ligated before the samples were retrotranscribed and again PAGE purified. Circularized complementary DNA (cDNA) templates were amplified with 12 PCR cycles, using Phusion polymerase (NEB). Following an additional native PAGE purification (8% TBE gel) step, libraries were quantified using the Qubit fluorometer, while the quality and average fragment size were estimated at the Bioanalyzer (High Sensitivity assay, Agilent). If not described otherwise, steps of the library generation were performed according to the mammalian ARTseq kit.

Barcodes were used to perform multiplex sequencing and create sequencing pools containing at least eight different samples and always an equal amount of both RNA and RPF libraries. We then sequenced sample pools on several lanes on the HiSeq 2000 platform using 50-bp sequencing chemistry to reduce barcode-, lane- or slide-related bias in the sequencing data.

To assess mRNA levels, we analysed a polyA-selected RNA-seq data set, termed ‘RNA-seq1', of the BN-*Lx* and SHR/Ola strains (five biological replicates)^31^. Adapters were ligated to random-primed cDNA with the SPRI bead system and sequencing was performed on a HiSeq 2000 instrument. To account for non-polyadenylated transcripts and for intra-strain variation in gene expression, we additionally generated RNA-seq libraries in parallel to ribosome profiling from identical tissue samples, omitting nuclease digestion, monosome purification as well as the initial PAGE purification step. The RNA was heat fragmented before adapter ligation. This data set is referred to as ‘RNA-seq2' in the manuscript. The integration of both data sets ensures that we can differentiate the three different modes of gene expression: forwarding, buffering and reinforcement (see Differential expression analysis). Translationally induced differences in gene expression can be investigated considering both expression levels of polyadenylated transcripts as well as total RNA levels.

### Sequencing data processing and quality control

Raw sequencing data were demultiplexed with the CASAVA 1.8 pipeline and the 3′-end adapter was removed using the FASTX-toolkit ( http://hannonlab.cshl.edu/fastx_toolkit/), retaining reads of 20 nt or longer post-clipping length: fastx_clipper -a 5′-AGATCGGAAGAGCACACGTCT-3′ -l 20 -n -v -Q33 followed by fastx_trimmer -Q33 -f 1.

We then created a custom bowtie2 (ref. 40) index to remove abundant sequences in ribosome data from further analyses. Fasta files of the rat mitochondrial genome and rRNA sequences were obtained from the Ensembl^41^ database. Rat transfer RNA (tRNA) sequences were obtained from the Genomic tRNA Database^42^ and were downloaded from http://gtrnadb.ucsc.edu/download.html. Clipped reads were then aligned against the custom bowtie2 index (Supplementary Fig. 1c) using standard parameters. Reads that could not be aligned were considered ‘clean' and used for further analyses: bowtie2 -L 20 --un clean.fastq.

We pinpoint translational control by identifying significantly differential read counts in Ribo-seq and RNA-seq data across strains and categorize differential gene expression events specific to either both or only one data set type. To avoid differences that arise through differing attributes of the sequencing data such as read length or sequencing depth, we matched the data before detecting strain-specific differences in read abundance. To avoid differences between RNA-seq and Ribo-seq methodologies that arise through mapping artefacts, we then trimmed all RNA-seq reads to 29 nt length before alignment: fastx_trimmer -l 29 -Q33. This is the most common length of Ribo-seq reads (Supplementary Fig. 1b).

To avoid mapping artefacts due to split-read alignment of the short reads, we defined splice junctions that were detected in at least four animals using TopHat^43^ using the full-length paired-end reads (2 × 100 bp) of RNA-seq1 (ref. 31). These junctions, in addition to splicing events as annotated by the Ensembl release 72 database^41^, were considered when mapping the trimmed RNA-seq and Ribo-seq data.

To prevent strain-specific mapping biases, we also infused genetic variation of either the BN-*Lx* or the SHR/Ola strains reported by Atanur *et al.*^20^ into rn5 genome. We then proceeded to map RPF and trimmed RNA libraries to their respective BN-*Lx-* or SHR/Ola- infused genomes and transcriptomes with TopHat2.0.8 (ref. 44), allowing for *de novo* splice junction detection and the following options: tophat --read-realign-edit-dist 0 -M.

We then, for each sample, counted the number of reads mapping uniquely to only one genomic position and that can be assigned to an annotated exon, not considering the mitochondrial chromosome. On the basis of this number, we randomly downsampled Ribo-seq and RNA-seq libraries for each biological replicate individually to improve comparability and avoid power issues while detecting expression differences introduced by variation in sequencing depth across technologies. For each animal, we matched and downsampled both RNA-seq and the Ribo-seq libraries to the size of the library with the lowest read counts within each animal (Supplementary Fig. 2).

All sequencing data sets were then matched in depth (effective depth of reads contributing to the analysis) and read length to ensure that exclusive characteristics of Ribo-seq or RNA-seq experiments reflect true biological processes and not technical properties of the different methods.

To assess the quality of the Ribo-seq data sets, we calculated the length-normalized, genome-wide average expression of the UTR and coding regions. For each library, we counted the number of reads mapping to either 5′UTR, Coding Sequence or 3′UTR and normalized it by the total length of the respective feature and the library size. Ribosome footprints of the heart and liver were distributed across gene bodies as expected^11^ and mainly covered the coding sequence of genes or to a lesser extent the 5′UTR. The 3′UTR was depleted of reads in the Ribo-seq data sets (Supplementary Fig. 1a). We also determined the position of read starts in the proximity of the start and stop codon in the Ribo-seq data. For all 20 Ribo-seq data sets, we observed clear triplet periodicity, distinctive for actively translating ribosomes, and a strong peak of 29-nt oligomers at 12 bp upstream of the start codon, indicating ribosomes located at the translation start site. To estimate sample-to-sample distances, we clustered all samples within each data set according to the Euclidean distances using the DESeq2 (refs 21, 45) package (Supplementary Fig. 2).

### Differential expression analysis

To quantify expression of genes, we first assigned uniquely mapping reads to their genomic feature (Ensembl database release 72 (ref. 41)) and treated spliced reads between exons as a single fragment using HTSeq: htseq-count --stranded=no --type=exon --idattr=gene_id. DESeq2 (refs 21, 45) with standard parameters was then used to deduce significant alterations in transcription and translation of cellular transcripts (Supplementary Fig. 3; Bonferroni adj. *P*≤0.01).

To determine the relationship of transcriptional and translational regulation, we defined three groups of genes: (i) RNA+RIBO: denotes forwarded significant differences in expression across strains that are detectable in both RNA-seq experiments as well as in the Ribo-seq data (Bonferroni adj. *P*≤0.01). Differential RNA expression of these genes is promoted to the translational level and has a direct effect on protein synthesis. (ii) RNA_only_: are buffered genes that do not exhibit significant inter-strain variation on the translational level but that were detected as differentially transcribed in both RNA-seq experiments (Bonferroni adj. *P*≤0.01). Translational regulation counteracts changes on the RNA level and buffers differences in between the strains. (iii) RIBO_only_: indicates reinforced and translationally induced strain-specific differences, which are exclusive to Ribo-seq and cannot be detected in either of the two RNA-seq experiments (Bonferroni adj. *P*≤0.01). Ribosome profiling can reveal differential usage of genes that is either not present or under-represented in RNA expression data sets.

To define differences between Ribo-seq and RNA-seq data that arise due to translational regulation, we performed RNA sequencing using two different methodologies: sequencing of rRNA-depleted total RNA (RNA-seq2) and polyadenylated transcripts (RNA-seq1).

RNA-seq2 serves as a technical control for Ribo-seq. The libraries were created in parallel from the identical tissue lysate with a procedure very similar to Ribo-seq. However, ribosomes occupy mostly processed transcripts, whereas total RNA-seq also contains nuclear RNA. Thus we also used RNA-seq1 (PolyA-selected RNA-seq) as a control data set to determine whether processed transcripts are differentially expressed on the RNA level.

For each data set, we also calculated fold changes of normalized read counts per gene for SHR/Ola over BN-*Lx*. RNA fold changes are the average of RNA-seq1 and RNA-seq2. To assess whether RNA-seq and Ribo-seq fold changes correlate significantly on a global scale, we computed the Pearson's correlation for both tissues (*P*<1e−4).

Comparing RNA-seq and Ribo-seq fold changes uncovers the extent of translational regulation for each gene. When differences on the RNA level induce equivalent changes in the ribosome occupancy, no translational regulation is present and strain-specific differences are promoted across regulatory layers. An increase or decrease of fold changes across data sets indicates translational reinforcement or buffering. To estimate the extent of translational regulation for the RNA_only_, RNA+RIBO and RIBO_only_ gene sets, we computed the slopes of three linear models:


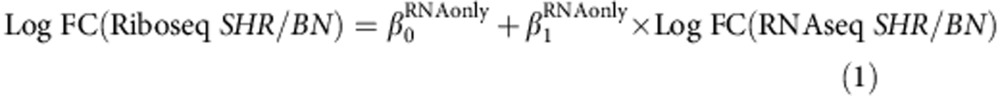



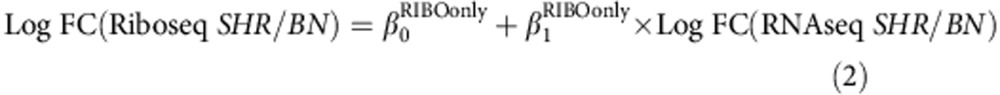



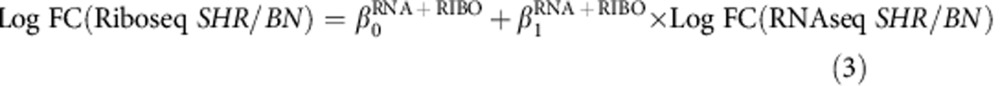


using the major-axis estimation method^46^. We then tested the null hypothesis of 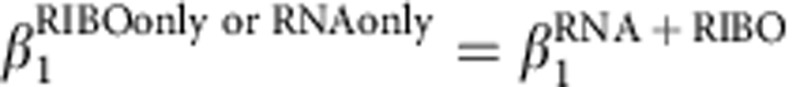 using the likelihood ratio statistic implemented in the smatr3 R package^25^.

### Proteome integration

Global protein abundances were estimated in the liver of BN-*Lx* and SHR/Ola using liquid chromatography-tandem mass spectrometry (LC-MS/MS) as reported by Low *et al.*^26^ We calculated the expression of genes based on RNA-seq (average of RNA-seq 1 and RNA-seq2) as well as on Ribo-seq data sets of both strains in the liver (fragments per kilobase per million reads mapped≥1) and correlated these values with MS-based proteomic data (all data were log transformed). Correlation between Ribo-seq and protein levels was higher than correlation between RNA-seq and protein levels in both strains (Supplementary Fig. 5). To assess whether our data support the expected model for the flow of genetic information from transcription, through translation, to protein levels, we tested for conditional independence using partial correlations^27^.

We consider the three random variables ‘RNA', ‘Ribo' and ‘protein'. For each strain we tested all pairs of the three variables ‘RNA', ‘Ribo' and ‘protein' for conditional independence, given the third variable. Intuitively, conditional independence between ‘RNA' and ‘protein' conditional on ‘Ribo' means that knowledge about ‘RNA' is irrelevant for knowledge about ‘protein' if we know ‘Ribo'. We assume that tuples (‘RNA', ‘Ribo', ‘protein') follow a multivariate Gaussian distribution. Then the conditional distribution of two variables, given the third, is a bivariate Gaussian distribution, with the partial correlation coefficient describing the relation between the two variables. In this model, conditional independence is equivalent to a zero partial correlation coefficient. Partial correlation coefficients can be obtained from the inverse of the covariance matrix or by correlating the residuals obtained by regressing each of the two variables against the third. Here we used the second approach and tested the significance of the partial correlation coefficient using the *t*-distribution^47^.

To test whether variation in ribosome occupancy—in the absence of differential RNA expression—is also reflected on the protein level, we investigated the regulation of 179 proteins in more detail. These proteins were found to be under translational regulation (RIBO_only_) in the liver and were also quantified by MS in both strains. Translationally induced strain-specific differences resulted in significant concordant changes (Wilcoxon–Mann–Whitney; *P*<1e−4) on the protein level (Fig. 2b).

### Translational efficiency

The TE score was calculated for the different gene groups (RNA_only_, RIBO_only_ and RNA+RIBO) as the ratio of normalized Ribo-seq reads in the CDS over normalized RNA-seq reads (average of RNA-seq1 and RNA-seq2) in the CDS, thus avoiding the length of UTRs to influence our calculation. Ribo-seq and RNA-seq data cover different parts of genes making it necessary to focus on the CDS, a region covered by both methodologies, when performing quantitative comparisons across both technologies.

### UTR length and SNP enrichment

We determined the length and position of UTRs as annotated by the Ensembl release 72 database^41^ and tested for differential length and SNP density using the Wilcoxon–Mann–Whitney method.

### Enrichment of KEGG pathways

We compared enrichment analyses based on gene sets differentially regulated according to either technology: RNA-seq (RNA+RIBO and RNA_only_) or Ribo-seq (RNA+RIBO and RIBO_only_). We identified a significant over-representation of KEGG^48^ pathways in differentially used genes for both Ribo-seq and RNA-seq data using webGestalt^49^. At least two genes for each pathway were required. Pathways had to be significantly (*P*_adjusted_≤0.01; Bonferroni) enriched according to a hypergeometric test. All pathways listed by the KEGG database (as of 21 March 2011) were tested for a significant over-representation of differentially expressed genes. Pathways were either detected using both methodologies (black), or exclusively enriched in either Ribo-seq (red) or RNA-seq (blue) data (Supplementary Data 3–5).

### miRNA expression

To test for differential miRNA expression, we analysed four biological replicates of each strain. Total RNA was extracted from the left ventricle tissue using the mirVana miRNA Isolation kit (Life Technologies). RNA was quantified and the quality assessed using Agilent's Bioanalyzer. To enrich for 15–35-bp small RNAs, 10 μg total RNA was purified on 15% denaturing PAA gel (7 M urea). Small RNA libraries were prepared using the SOLiD Total RNA-Seq kit (Life Technologies) following the manufacturer's guidelines. After adapter ligation, cDNA was purified using the MinElute PCR Purification kit (Qiagen) and size selected on a 10% TBE-urea gel. cDNA fragments were then amplified using barcoded primers for multiplex sequencing. The final cDNA libraries were quantified using the Qubit fluorometer, library sizes were assessed with the DNA 1,000 kit (Agilent) and sequencing was carried out on a SOLiD 3 system.

### Genome mapping and miRNA quantification

Adapter-clipped reads were mapped against rn4 with Bowtie^50^ (Bowtie options: -l 17 -a --m 5 -n 2 --best --strata -C -f) to accurately quantify annotated rat miRNAs^51^,^52^,^53^. We assessed the expression of 680 annotated mature miRNAs. We performed a quantile-based scaling to normalize read counts as described previously^54^. Normalized and rounded miRNA counts were then compared between BN-*Lx* and SHR/Ola using a generalized linear model with the quasi-Poisson family. The FDR was controlled using the Benjamini–Hochberg procedure^55^. We then assessed whether differential miRNA expression was associated preferentially with transcriptional or post-transcriptional control of protein synthesis by comparing the union of the target sets of all differentially expressed miRNAs with the classification of genes based on differential translation and transcription. We found that genes with variation in translational regulation (RIBO_only_) were more often predicted targets^56^ of differentially expressed miRNAs (FDR<0.05) than differentially expressed (RNA+RIBO) genes (Supplementary Table 5) using Fisher's exact test in each tissue separately. Finally, we combined the results from both tissues using the Fisher method^57^.

### eQTL analysis

To identify loci that determine RNA expression levels and the respective target genes, we previously performed an eQTL study for both tissues based on RNA-seq data of the HxB/BxH recombinant inbred panel^31^. Briefly, we sequenced the RNA of one animal for each of the 30 recombinant inbred strains, each on one lane of a HiSeq 2000 Illumina instrument with TruSeq 2 × 100-bp paired-end chemistry. Reads were aligned to the reference genome RGSC 3.4 using TopHat v 1.2.0 (ref. 43). Gene expression levels were estimated by counting reads within gene bodies. Gene expression levels were normalized using a quantile-based scaling method^54^. QTLs were identified by trait–marker regression using a negative binomial regression model^58^ similar to the model used for differential analysis in DESeq. The genetic map consisted of 1,384 non-redundant SNP genotype profiles. For each pair of trait *i* and marker *j*, we computed the likelihood ratio statistic of the full model containing the genotype variable against a null model containing only an intercept term. To determine the significance of likelihood ratio statistic scores while accounting for linkage disequilibrium, we used a permutation strategy. Finally, we adjusted the QTL *P* values for multiple testing using the Benjamini–Hochberg method^55^.

We then compared the classification of genes based on differential translation and transcription to the set of genes that have eQTL using the *χ*^2^-test. As expected, we found a strong enrichment of eQTLs for differentially expressed genes on the RNA level in the founder strains (Supplementary Table 2). Genes that have an eQTL in either tissue were mostly detected as being also differential using Ribo-seq, which indicates that genetically induced variation in RNA expression is carried forward to translation (Fig. 3a,b). Genes predicted to be mostly under translational control (RIBO_only_) were not enriched for eQTLs, thus representing a layer of regulation not captured by RNA expression-based linkage studies.

### Motif analysis

To determine whether the binding sites of expressed RNA-RBPs were preferentially altered by SNPs in 3′UTRs of translationally regulated (RIBO_only_) genes as compared with SNPs in 3′UTR of genes that were regulated on both levels (RNA+RIBO), we first obtained 50 bp of flanking sequences for all SNPs located in all 3′UTR regions subject to the integrated analysis in each tissue. For each position weight matrix from the Hughes RBP database^59^, we scored the difference of binding affinities of the reference and alternative alleles using the sTRAP method^60^. We then classified the SNPs into two classes: SNPs in RIBO_only_ genes and SNPs in RNA+RIBO genes. We tested for each position weight matrix whether the absolute value of the log ratio of binding *P* values was larger in the RIBO_only_ group compared with the RNA+RIBO group using a one-sided Wilcoxon–Mann–Whitney test. Finally, we adjusted these *P* values for multiple testing using the Benjamini–Hochberg method^55^.

### Quantitative real-time PCR

Total RNA was extracted from pulverized left ventricle or liver tissue of both strains using TRIzol (Life Technologies). After DNase I (Ambion) treatment at 37 °C for 20 min, RNA was purified using the RNeasy Mini kit (Qiagen). First-strand cDNA synthesis was performed using random primers and Superscript II reverse transcriptase (Life Technologies).

To estimate RNA expression of genes, quantitative PCRs were performed using the Power Sybr Green mix (Applied Biosystems) and primers listed in Supplementary Table 7. Quantitative real-time PCRs were run on the ViiA7 Real-Time PCR System (Applied Biosystems).

We included five biological replicates for each strain and tissue and performed four technical replicates per sample. Expression levels were calculated using the ΔΔ^*C*t^ method and normalized to *Polr2a* (heart samples) or *Tbp* (liver samples). Differential RNA expression was tested using the nonparametric Mann–Whitney test.

### Western blotting

Protein extracts were obtained by lysing pulverized left ventricle or liver tissue in RIPA buffer (10 mM Tris-HCl pH 7.6, 140 mM NaCl, 1 mM EDTA, 0.1% SDS, 0.1% sodium deoxycholate and 1% Triton X-100) supplemented with a protease inhibitors cocktail (Roche) on ice for 30 min. Samples were centrifuged at 20,000*g* for 10 min at 4 °C. Total protein concentration was estimated using the BCA protein assay kit (Pierce). Proteins were resolved on 4–12% Bis-Tris Novex gels (Life Technologies) under reducing conditions and then transferred on polyvinylidene difluoride membranes (Millipore). To block for unspecific sites, membranes were incubated with 5% non-fat dry milk in PBS-T (1 × PBS, 0.01% Tween20). Primary antibody incubations in 5% non-fat dry milk in PBS-T were performed overnight at 4 °C. The following primary antibody dilutions were used: 1:100 Ctps1 (ab133743, Abcam), 1:1,000 Fads1 (EPR6898, Abcam), 1:200 Fes (sc-7671, Santa Cruz Biotechnology), 1:1,000 Gja1 (#3512, Cell Signaling), 1:2,000 Maoa (ab126751, Abcam), 1:500 Mrpl48 (H00051642-A01, Abnova), 1:200 Myh6 (sc-16876, Santa Cruz Biotechnology), 1:1,000 Myh7 (ab11083, Abcam), 1:1,000 Ppia (ab41684, Abcam) and 1:2,000 Vinculin (V9264, Sigma) as the loading control.

Secondary horseradish peroxidase-conjugated antibodies anti-goat (sc-2020, Santa Cruz Biotechnology), anti-rabbit (W401B, Promega) or anti-mouse (W4021, Promega) were used at a dilution of 1:2,000. Membranes were developed using the chemiluminescent detection method. Results are summarized in Supplementary Fig. 6 and full-size images are shown in Supplementary Fig. 8.

## Additional information

**Accession codes:** Data are available via the European Nucleotide Archive (ENA; http://www.ebi.ac.uk/ena/data/view/PRJEB7498) under accession number (PRJEB7498).

**How to cite this article:** Schafer, S. *et al.* Translational regulation shapes the molecular landscape of complex disease phenotypes. *Nat. Commun.* 6:7200 doi: 10.1038/ncomms8200 (2015).

## Supplementary Material

Supplementary Figures and Tables Supplementary Figures 1-8 and Supplementary Tables 1-7

Supplementary Data 1 Differentially expressed genes in the rat heart. Comparison of gene expression levels between SHR/Ola and BN-Lx rat strains using both RNA-seq and Ribo-seq data reveals genes under three different modes of gene expression regulation in the heart. Genes differentially expressed on the RNA level were either buffered through translation (blue; RNAonly) or the variation in expression was also detected in the translational level (black; RNA+RIBO). We also detected hundreds of genes under translational control that were not regulated on the RNA at genome-wide significance (red; RIBOonly).

Supplementary Data 2 Differentially expressed genes in the rat liver. Comparison of gene expression levels between SHR/Ola and BN-Lx rat strains using both RNA-seq and Ribo-seq data reveals genes under three different modes of gene expression regulation in the liver. Genes differentially expressed on the RNA level were either buffered through translation (blue; RNAonly) or the variation in expression was also detected in the translational level (black; RNA+RIBO). We also detected hundreds of genes under translational control that were not regulated on the RNA at genome-wide significance (red; RIBOonly).

Supplementary Data 3 KEGG enrichment analysis for differentially used genes in heart. All differentially expressed genes detected in either Ribo-seq or RNA-seq datasets were tested for significant (Hypergeometric test, Bonferroni corrected P = 0.01) enrichment of KEGG6 pathways using webGestalt7. The colors indicate wether an enrichment was detected with both technologies (black), only considering Ribo-seq (red) or only with RNA-seq (blue).

Supplementary Data 4 KEGG enrichment analysis for differentially used genes in liver. All differentially expressed genes detected in either Ribo-seq or RNA-seq datasets were tested for significant (Hypergeometric test, Bonferroni corrected P = 0.01) enrichment of KEGG6 pathways using webGestalt7. The colors indicate wether an enrichment was detected with both technologies (black), only considering Ribo-seq (red) or only RNA-seq (blue) data.

Supplementary Data 5 Detailed KEGG pathways enriched for differentially expressed genes in the liver and heart between SHR/Ola and BN-Lx. All differentially expressed genes detected by either Ribo-seq or RNA-seq datasets were tested for significant (Pcorrected = 0.01, Bonferroni) enrichment of KEGG6 pathways using webGestalt7. Individual genes that are members of each pathway are listed for each analysis: Heart RNA-seq; Heart Ribo-seq; Liver RNA-seq and Liver Ribo-seq.
