# Supplementary material for: Translational regulation shapes the molecular landscape of complex disease phenotypes
Source: Nat Commun. 2015 May 26;6:7200. doi: 10.1038/ncomms8200 (PMC4455061; doi:10.1038/ncomms8200)
Supplement: Supplementary Figures and Tables — Supplementary Figures 1-8 and Supplementary Tables 1-7 [file ncomms8200-s1.pdf]

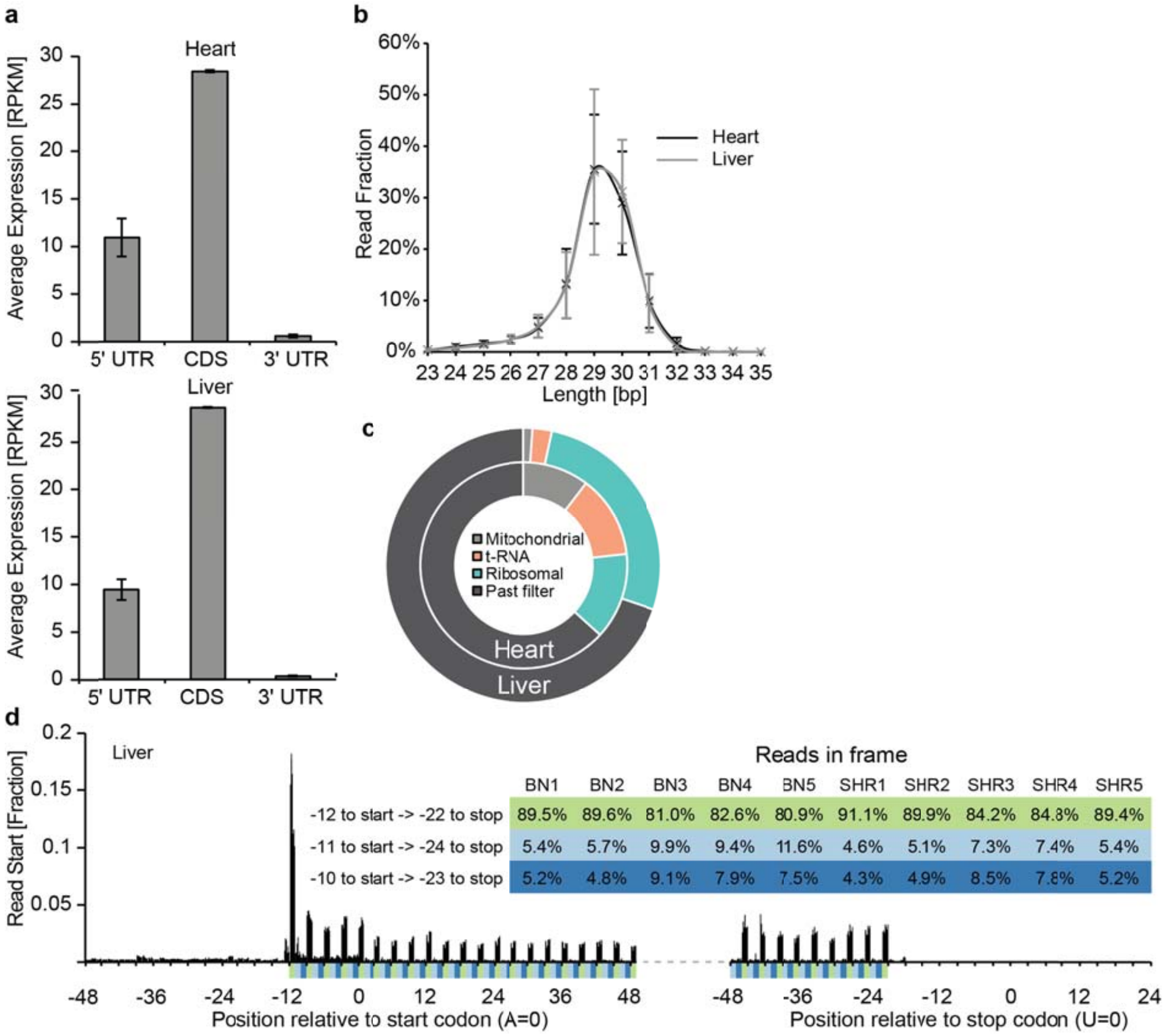


**Supplementary Figure 1.** General attributes and quality control for RPF libraries of frozen tissues. (**a**) Read distribution across gene bodies is shown for 10 libraries (5x SHR/Ola; 5x BN-*Lx*) for each tissue. Reads covering 5'UTR, CDS or 3'UTR were normalized to feaure length and library depth to calculate the average "reads per kilobase per million reads" RPKM. Ribosome footprints are mainly found in the coding region of transcripts and to a lesser extent in the leader sequence (5' UTR), while the average RPKM for the 3' UTR in both tissues is less than 0.5. (**b**) Average read length distribution of 10 heart (black) and 10 liver (grey) Ribo-seq libraries. Ribosomes protect mostly 29 nt on transcripts. (**c**) Pie charts illustrate the average of mitochondrial, tRNA and rRNA read fractions for heart and liver datasets. The "past filter" share denotes the reads used for downstream analysis. (**d**) Periodicity profile of RPFs in hepatic tissue at a subcodon resolution. The plot shows, for each sample, the number of RPF reads' 5' termini (read start) aligning in 48 nt windows around start and stop codons. Only reads with a length of 29 nt were considered. Ribosomes located with the P site at the start codon protected fragments starting at 12 bp upstream of the AUG codon. Ribosomes detach from transcripts once the A site of the ribosome reaches the stop codon. For each library, the fraction or reads covering each frame is shown. The majority of ribosomes are located on the codons of the open reading frame of protein coding genes. Error bars indicate s.d..

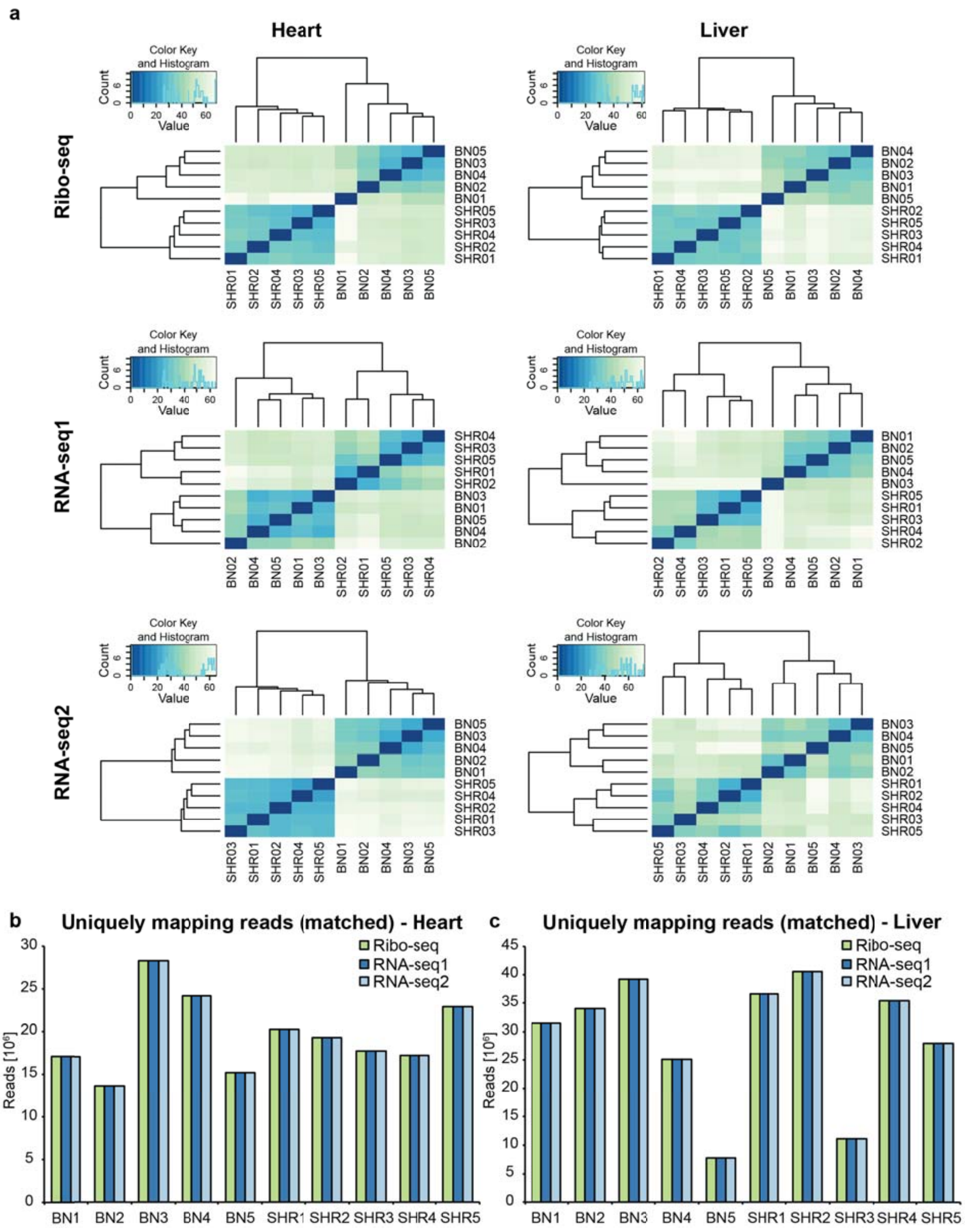


**Supplementary Figure 2.** Sample clustering and coverage. (**a**) Heatmaps show sample-to-sample Euclidean distance in-between replicates and strains according to DESeq2[1] for RNA-seq and Ribo-seq datasets. (**b, c**) The number of reads mapping to one unique position in the genome and located in an exon is plotted for each biological replicate in both tissues. To ensure expression differences unique to a certain technique are not influenced by library sizes, we matched the number of reads mapping uniquely and located within an exon for both tissues before estimating gene expression levels.

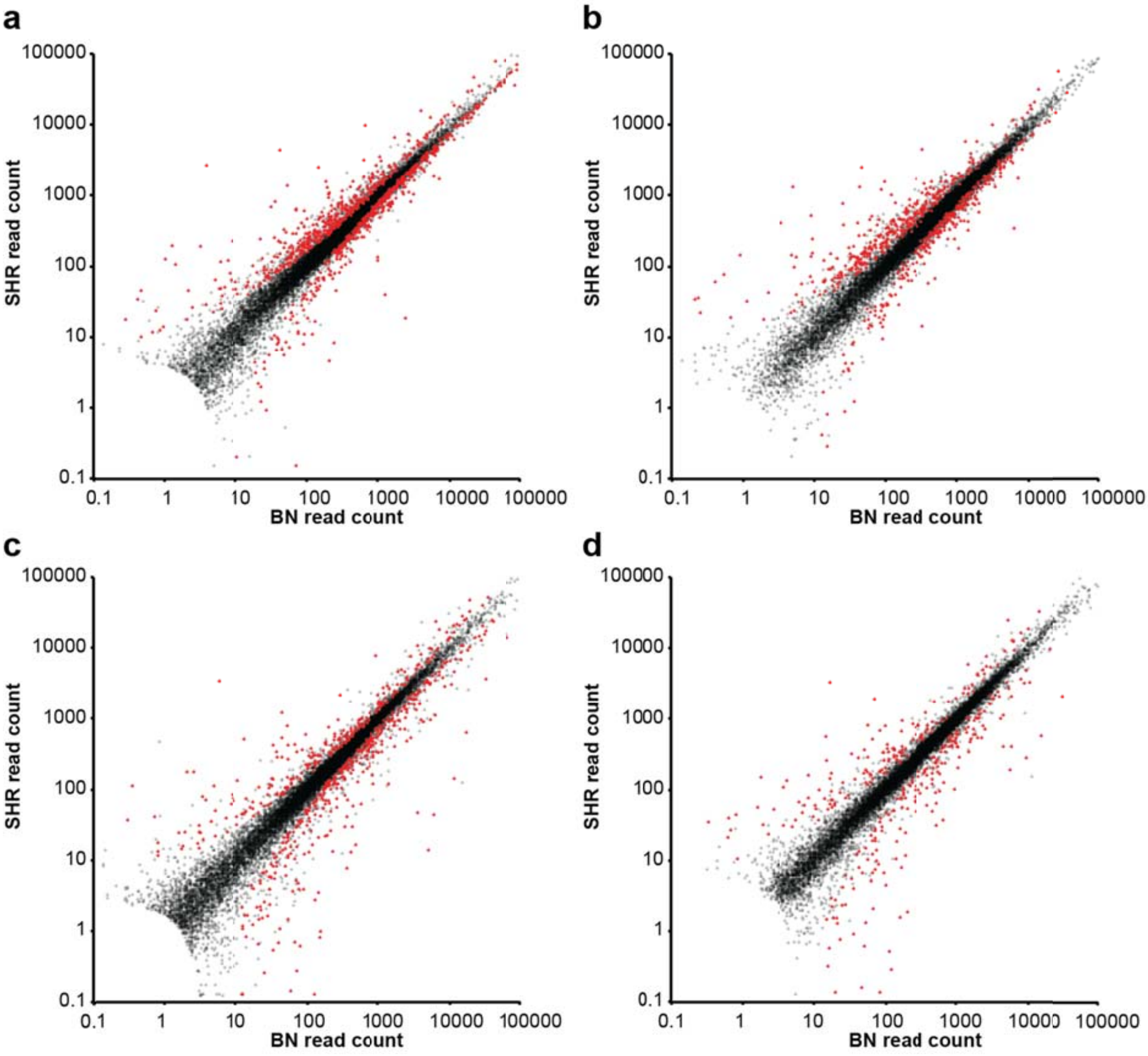


**Supplementary Figure 3.** Scatter plots of pairwise RNA-seq and Ribo-seq comparisons. (**a**) Heart Ribo-seq, (**b**) Heart RNA-seq; (**c**) Liver Ribo-seq and (**d**) Liver RNA-seq gene-based counts between SHR/Ola and BN-*Lx* rat strains. The red color indicates significantly different genes in-between the strains according to DESeq2 (Bonferroni correction for multiple testing, FDR $\leq$ 0.01). For RNA expression, we only considered genes that were detected in both RNA-seq experiments and plotted the average expression across RNA-seq1 and RNA-seq2.

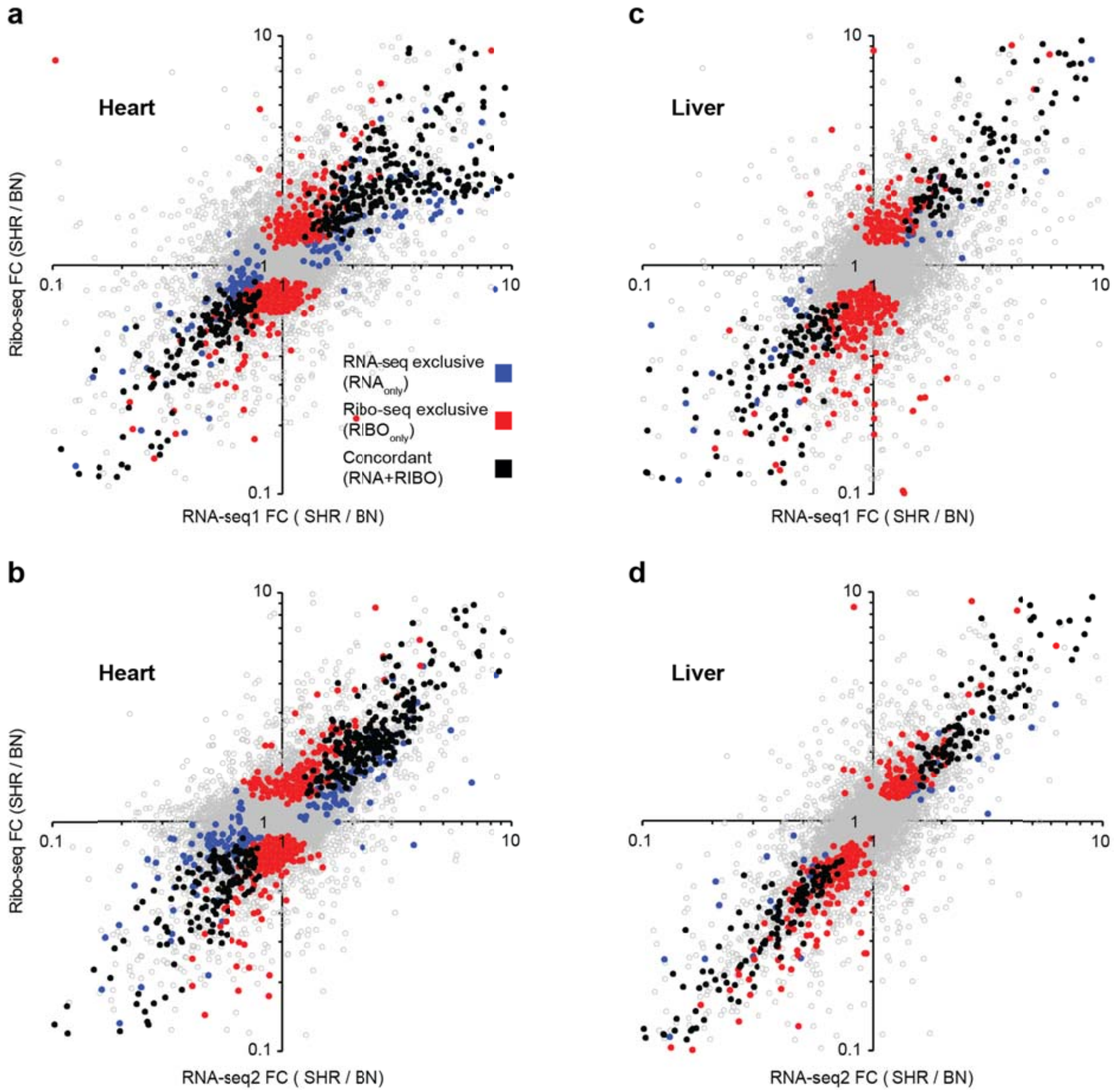


**Supplementary Figure 4.** Comparison of fold changes between Ribo-seq and RNA-seq datasets. (**a-d**) Fold change comparisons of Ribo-seq and RNA-seq count data for buffered ($RNA_{only}$), forwarded (RNA+RIBO) and reinforced ($RIBO_{only}$) genes in the heart and the liver between the SHR/Ola and BN-*Lx* rat strains. (**a**) Heart Ribo-seq fold-changes compared to RNA-seq1 (polyA+ RNA-seq) data; (**b**) Heart Ribo-seq fold-changes compared to RNA-seq2 (total RNA-seq) data; (**c**) Liver Ribo-seq fold-changes compared to RNA-seq1 (polyA+ RNA-seq) data and (**d**) Liver Ribo-seq fold-changes compared to RNA-seq2 (total RNA-seq) data. Gene classes indicated for each tissue were defined as described in the supplementary material.

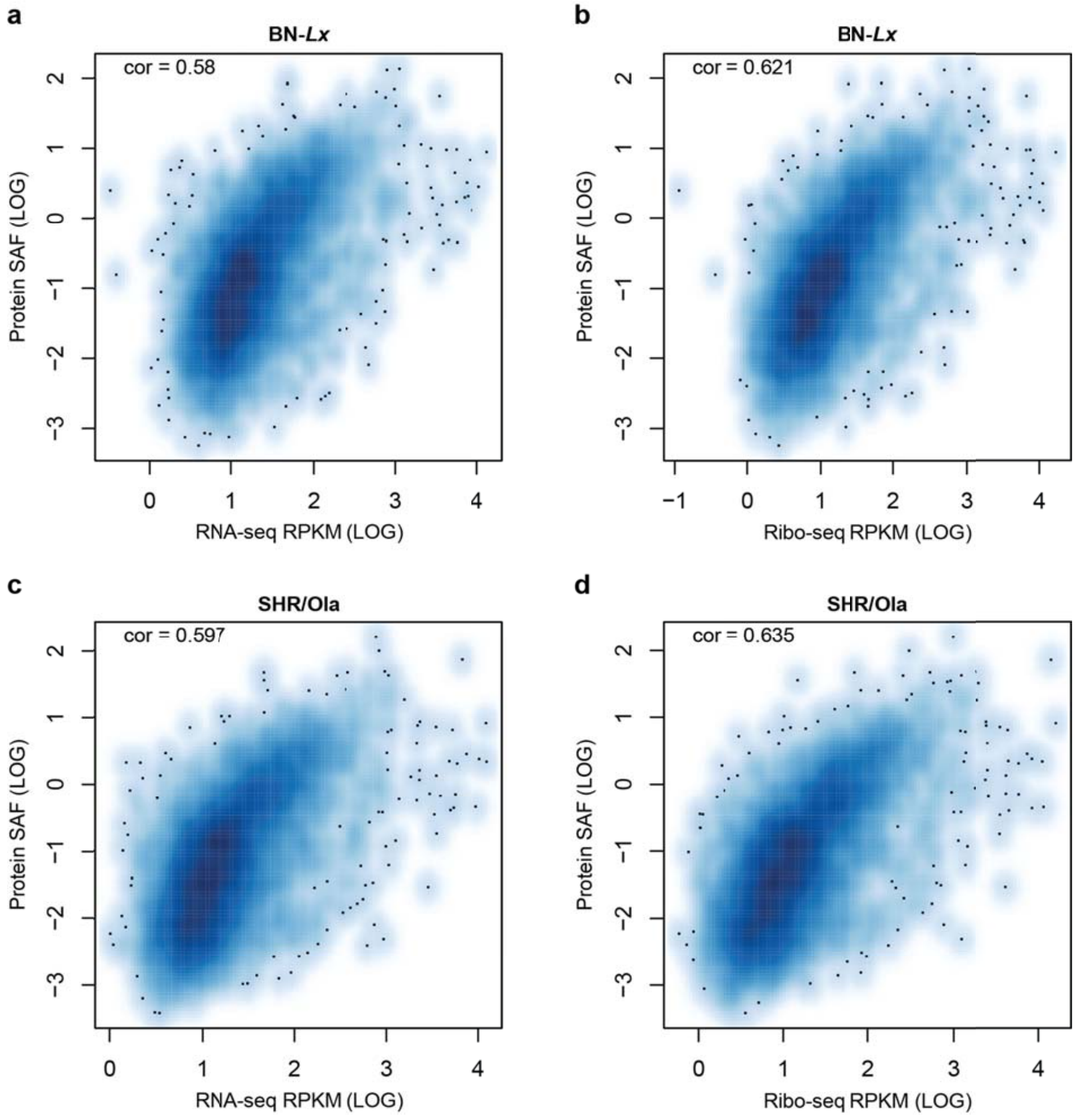


**Supplementary Figure 5.** Genome-wide correlation of RNA, RPF and protein abundance. Protein abundance in the liver as measured by mass spectrometry[2] is plotted against RNA-seq (BN-*Lx*: **a**, SHR/Ola: **c**) and Ribo-seq (BN-*Lx*: **b**, SHR/Ola: **d**) based RPKM values. In agreement to what has been previously reported in the literature[3], absolute protein levels show a better correlation with ribosome profiling data than with RNA-seq on the genome-wide scale.

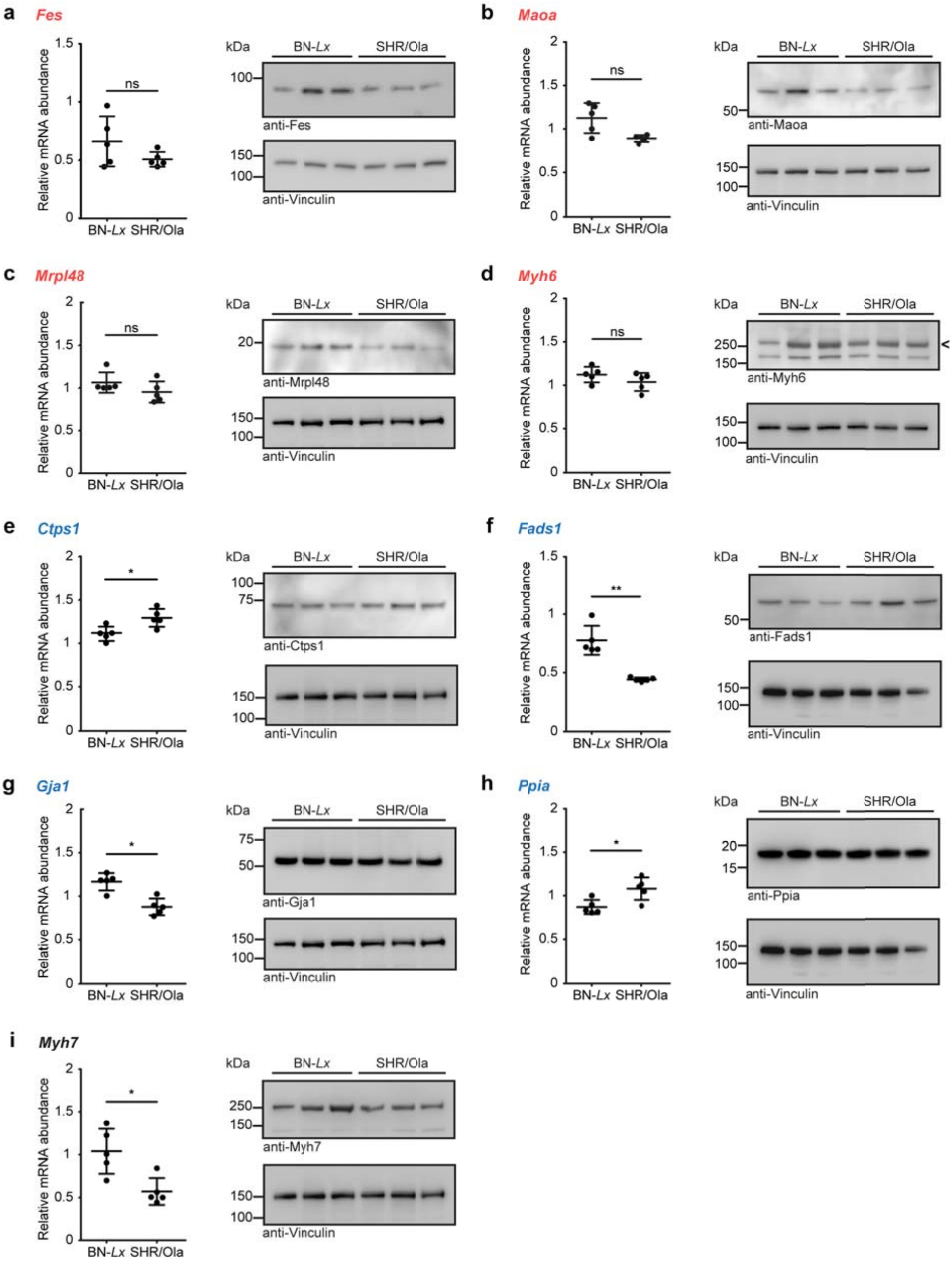


**Supplementary Figure 6.** Experimental validation of different modes of gene expression regulation. Genes belonging to each of the categories described in the manuscript ($RIBO_{only}$ in red, $RNA_{only}$ in blue, RNA+RIBO in black) were validated by qRT-PCR and western blotting. For each gene, qRT-PCR results are shown on the left (error bars indicate s.d.) and western blots on the right. **(a-d)** *Fes* (liver), *Maoa*, *Mrpl48* and *Myh6* (heart), which were classified as $RIBO_{only}$ genes, show differences on the protein level in-between the strains in the absence of significant RNA

expression differences (Mann-Whitney test). **(e-h)** *Ctps1*, *Fads1*, *Gja1* and *Ppia* (heart) belong to the $RNA_{only}$ group and quantitation by qRT-PCR shows significant differences (Mann-Whitney test, $P < 0.05$) at the RNA level between the strains, whereas comparable protein levels are detected by western blotting. **(i)** *Myh7* (heart) is classified as a RNA+RIBO gene and shows differences between BN-*Lx* and SHR/Ola both at RNA and protein levels.
In some cases the same membrane was used to detect two proteins of interest, after stripping. Therefore, the pairs Gja1-Mrpl48, Ctps1-Myh7, Ppia-Fads1 share the same loading controls.

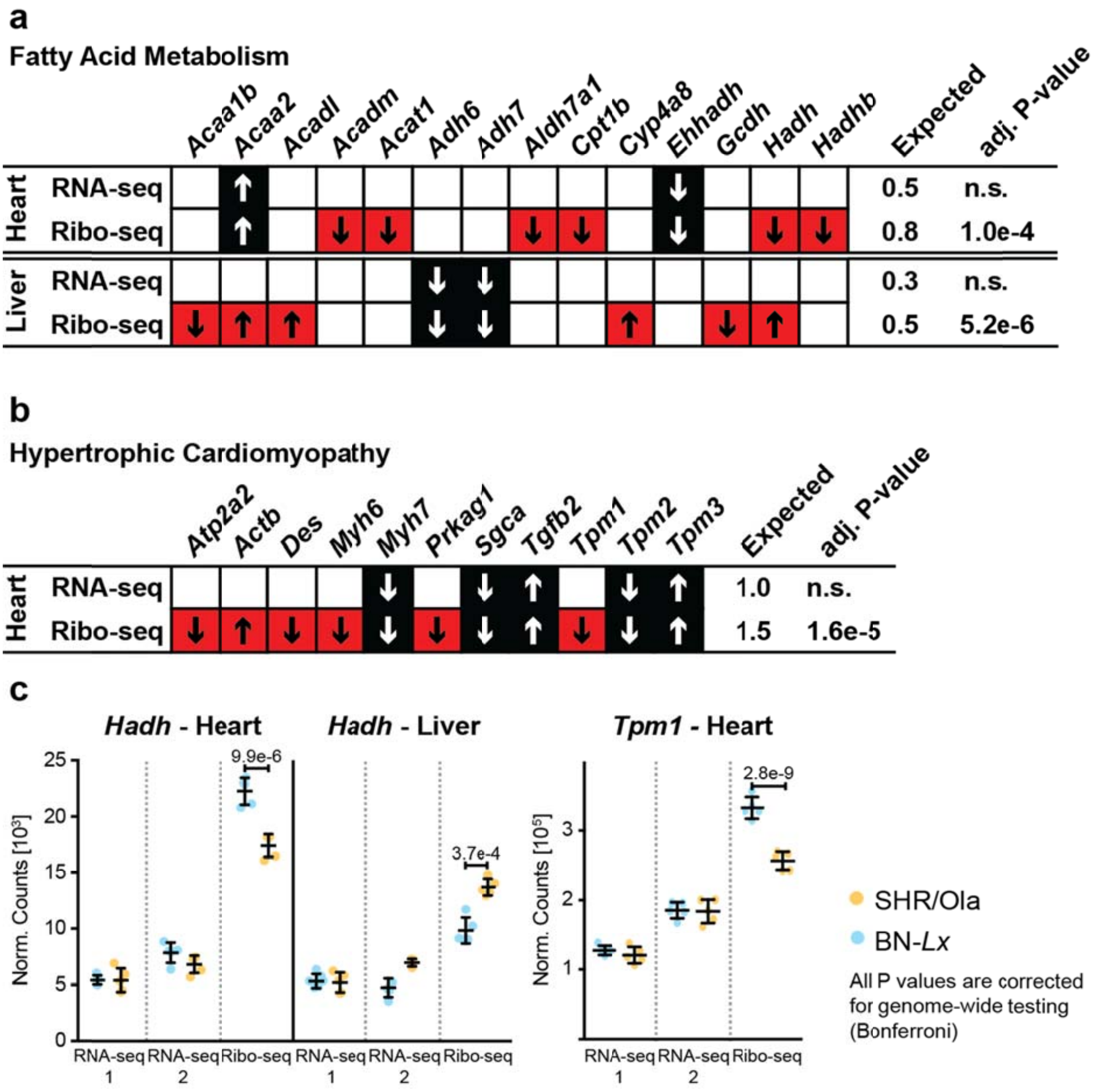


**Supplementary Figure 7.** Translational and transcriptional regulation of (**a**) fatty acid and (**b**) cardiomyopathy pathways (KEGG). Arrows indicate either up- or downregulation in the SHR. All differences detected on the RNA level for these metabolic pathways were confirmed to lead to changes in protein synthesis (black; RNA+RIBO). The majority of genes are regulated on the translational level between BN and SHR (red) and can only be detected as being differentially expressed using ribosome profiling techniques. (**c**) Read counts of *Hadh* and *Tpm1* across three different sequencing strategies (Ribosome profiling (Ribo-seq), polyA+ RNA sequencing (RNA-seq1) and total RNA sequencing (RNA-seq2)) visualize how novel and reinforced strain-specific differences in gene usage can be effectively revealed by Ribo-seq in both tissues. DESeq2 based P-values are corrected for genome-wide testing (Bonferroni, FDR ≤ 0.01). Error bars indicate s.d..

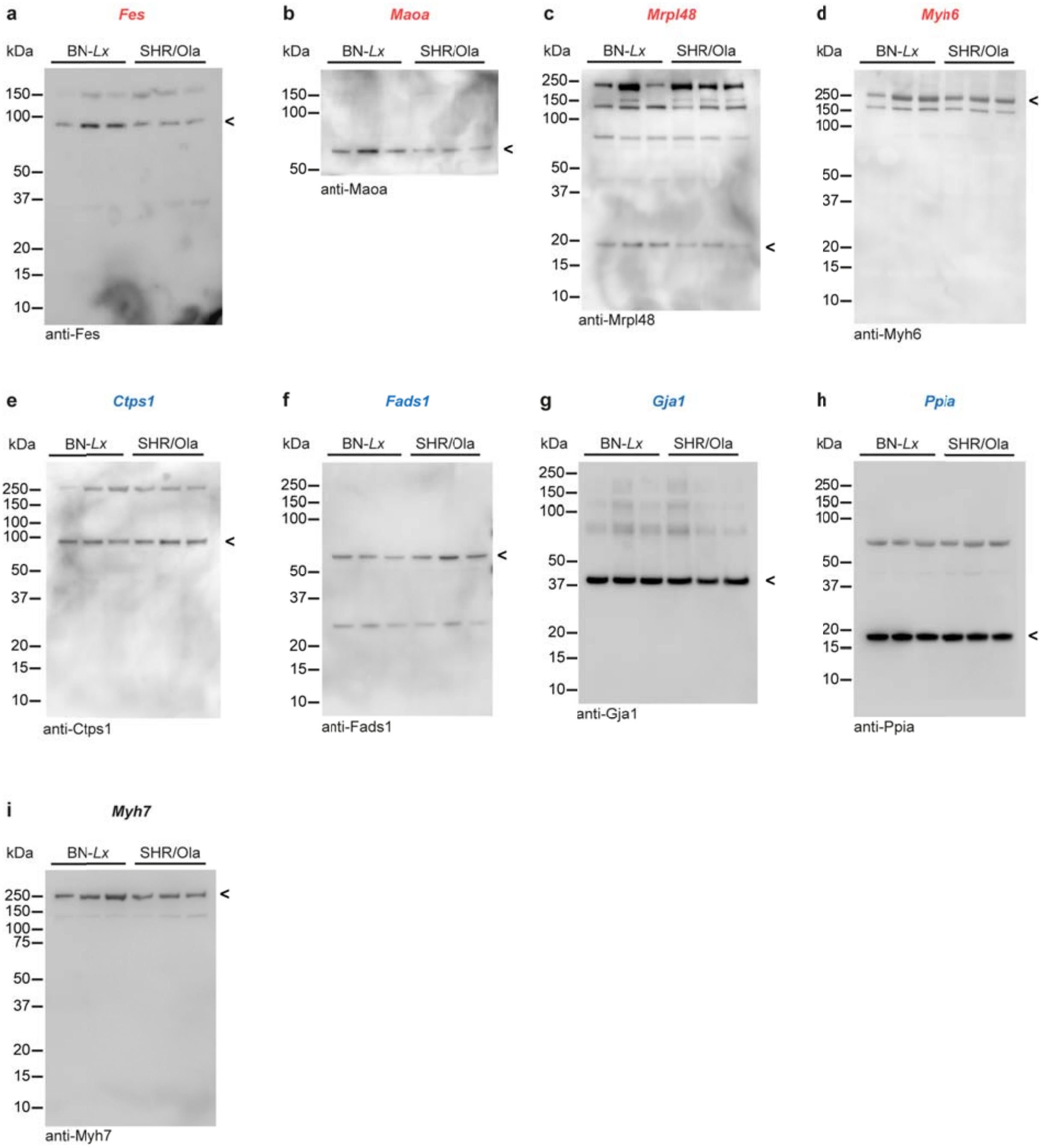


**Supplementary Figure 8.** Full size western blots corresponding to cropped images shown in **Supplementary Figure 6**. Gja1-Mrpl48, Myh7-Ctps1 and Fads1-Ppia were detected on the same membranes after adequate stripping procedures.

**Supplementary Table 1.** Partial correlation analysis of RNA-seq, Ribo-seq and protein levels. Using data of each strain separately**,** each pair of variables is tested for conditional independence given the third variable as denoted in the column “conditional independence” using partial correlation. The null hypothesis of zero partial correlation coefficients is tested using the t-statistic (column “t”) with the corresponding degrees of freedom (“df”) (see methods).

<table>
<tr><th>Strain</th><th>Conditional Independence</th><th>Partial Correlation</th><th>t</th><th>df</th><th>p</th></tr>
<tr><td>BN</td><td>(Ribo ~ Protein | RNA)</td><td>0.27</td><td>19.71</td><td>4757</td><td><2.2e-16</td></tr>
<tr><td>BN</td><td>(RNA ~ Protein | Ribo)</td><td>-0.03</td><td>-1.87</td><td>4757</td><td>0.06</td></tr>
<tr><td>BN</td><td>(RNA ~ Ribo | Protein)</td><td>0.92</td><td>156.93</td><td>4757</td><td><2.2e-16</td></tr>
<tr><td>SHR</td><td>(Ribo ~ Protein | RNA)</td><td>0.27</td><td>19.32</td><td>4757</td><td><2.2e-16</td></tr>
<tr><td>SHR</td><td>(RNA ~ Protein | Ribo)</td><td>-0.02</td><td>-1.29</td><td>4757</td><td>0.20</td></tr>
<tr><td>SHR</td><td>(RNA ~ Ribo | Protein)</td><td>0.92</td><td>159.00</td><td>4757</td><td><2.2e-16</td></tr>
</table>

**Supplementary Table 2.** Overlap of strain-specific gene usage and eQTL data. Linkage analysis of the HxB/BxH RI panel[4] using RNA-seq data derived from BN-*Lx* and SHR/Ola reveals RNA expression differences under genetic control. We then compared this eQTL data with all three modes of gene expression regulation in-between the parental strains. Genes with expression differences in the parental strains ($RNA_{only}$; RNA+RIBO) were enriched for eQTLs. Most gene expression traits on the RNA level (eQTLs) were found to alter protein synthesis rates (RNA+RIBO) in the parental strains. Only few eQTLs were buffered in the translational level (eQTL and $RNA_{only}$). As expected, strain-specific usage of $RIBO_{only}$ genes was not significantly enriched for RNA expression traits since the gene expression for these genes is regulated mostly on the translational level only (eQTL and $RIBO_{only}$).

| | | Strain-Specific Differences | | | |
|---|---|---|---|---|---|
| | **eQTL** | **$RNA_{only}$** | **RNA+RIBO** | **$RIBO_{only}$** | **n.s** |
| **Heart** | FALSE | 167 | 322 | 486 | 9211 |
| | TRUE (EXP) | 24 (4) | 119 (10) | 12 (11) | 83 |
| | TRUE / EXP | 5.6 | 11.9 | 1.1 | |
| **Liver** | FALSE | 39 | 199 | 343 | 11921 |
| | TRUE (EXP) | 13 (1) | 93 (3.2) | 11 (3.9) | 24 |
| | TRUE / EXP | 21 | 29 | 2.8 | |

**Supplementary Table 3.** Motifs of RNA binding proteins enriched for genetic variation in differentially translated genes across strains. We determined all motifs of RNA binding proteins[5] in the 3'UTR of genes that were under translational regulation ($RIBO_{only}$) in either heart or liver tissue between both rat strains. The motifs of 8 RNA binding proteins were more often mutated between the SHR/Ola and BN-*Lx* strains than expected (one-sided Wilcoxon-Mann-Whitney, corrected using the Benjamini-Hochberg method).

| CISBP-RNA Database ID | Ensembl ID | Gene Symbol | P Value | Corrected P Value |
|---|---|---|---|---|
| T41133_0.6 | ENSRNOG00000020271 | *Tial1* | 0.0012 | 0.0259 |
| T41136_0.6 | ENSRNOG00000020689 | *Cpeb3* | 0.0011 | 0.0259 |
| T41220_0.6 | ENSRNOG00000033169 | *Cpeb4* | 0.0011 | 0.0259 |
| T41110_0.6 | ENSRNOG00000016813 | *Tia1* | 0.0012 | 0.0259 |
| T41117_0.6 | ENSRNOG00000017405 | *Raly* | 0.0008 | 0.0259 |
| T40997_0.6 | ENSRNOG00000000702 | *Sart3* | 0.0024 | 0.0334 |
| T41142_0.6 | ENSRNOG00000021181 | *Sf3b4* | 0.0019 | 0.0334 |
| T41078_0.6 | ENSRNOG00000011621 | *D4ACR0_RAT* | 0.0034 | 0.0413 |

**Supplementary Table 4.** Expression levels of RNA binding proteins in heart and liver. The motifs of these RNA binding proteins are enriched for genetic variation in differentially translated genes across strains (**see Supplementary Table 3**). We calculated their RPKM value (average of 5 biological replicates) based on Ribo-seq data to test whether they were also translated in heart and liver tissue. If present in the tissue, they can potentially contribute to translational regulation between strains through *cis*-regulatory variation in their binding sites. We did not detect significant differential transcription or translation of these genes in-between strains.

| **Ensembl ID** | **Gene Symbol** | **Heart BN-*Lx* [RPKM]** | **Heart SHR/Ola [RPKM]** | **Liver BN-*Lx* [RPKM]** | **Liver SHR/Ola [RPKM]** |
|---|---|---|---|---|---|
| ENSRNOG00000020271 | *Tial1* | 40.4 | 41.4 | 20.1 | 25 |
| ENSRNOG00000020689 | *Cpeb3* | 16.2 | 12.9 | 3.6 | 3.6 |
| ENSRNOG00000033169 | *Cpeb4* | 23.2 | 26.3 | 10.8 | 12.9 |
| ENSRNOG00000016813 | *Tia1* | 14.7 | 13.7 | 0.9 | 0.9 |
| ENSRNOG00000017405 | *Raly* | 219.8 | 229.0 | 91.7 | 88.7 |
| ENSRNOG00000000702 | *Sart3* | 9.1 | 10.9 | 3.8 | 4.4 |
| ENSRNOG00000021181 | *Sf3b4* | 17.8 | 22.3 | 12.1 | 12.4 |
| ENSRNOG00000011621 | *D4ACR0_RAT* | 0.8 | 1.1 | 1.5 | 1.3 |

**Supplementary Table 5.** Differential miRNA expression and translational regulation. We determined differential miRNA expression for heart and liver tissues in-between rat strains. Differentially transcribed genes (RNA+RIBO) are not as strongly enriched for targets of differential miRNAs than genes under translational control (chi squared test, method Fisher meta P = 0.008).

| | Strain-Specific Differences | | | |
|---|---|---|---|---|
| | **ΔmiRNA Target** | **RNA+RIBO** | **$\mathbf{RIBO_{only}}$** | |
| **Heart** | FALSE | 224 | 207 | |
| | TRUE (EXP) | 217 (239) | 291 (269) | meta P = 0.008 |
| **Liver** | FALSE | 186 | 208 | |
| | TRUE (EXP) | 106 (114) | 146 (138) | |

**Supplementary Table 6.** GWAS candidate genes primarily under translational control ($RIBO_{only}$) in heart or liver in the SHR/Ola model for complex traits.

| Gene Symbol | Human ID | Orthologue Rat ID | Tissue | Trait | GWAS |
|---|---|---|---|---|---|
| *ACAA2* | ENSG00000167315 | ENSRNOG00000013766 | Liver | HDL cholesterol | 8 |
| *ACADL* | ENSG00000115361 | ENSRNOG00000012966 | Liver | Metabolite levels | 9,10 |
| *ACADM* | ENSG00000117054 | ENSRNOG00000009845 | Heart | Metabolite levels<br>Metabolic traits | 10,11 |
| *ACOT7* | ENSG00000097021 | ENSRNOG00000010580 | Heart | QT interval | 12 |
| *ADH5* | ENSG00000197894 | ENSRNOG00000033854 | Liver | HDL cholesterol<br>Obesity-related traits | 13 |
| *AQP9* | ENSG00000103569 | ENSRNOG00000015949 | Liver | Metabolite levels | 14 |
| *CCDC141* | ENSG00000163492 | ENSRNOG00000012580 | Heart<br>Liver | Blood pressure<br>Heart rate | 15,16 |
| *COL4A1* | ENSG00000187498 | ENSRNOG00000016281 | Heart | Coronary heart/artery disease<br>Obesity-related traits | 17–19 |
| *COL4A2* | ENSG00000134871 | ENSRNOG00000023972 | Heart | Coronary artery calcification<br>Coronary heart/artery disease | 17,19,20 |
| *CPN1* | ENSG00000120054 | ENSRNOG00000013439 | Liver | Liver enzyme levels | 21 |
| *ELMO1* | ENSG00000155849 | ENSRNOG00000018726 | Heart | QT interval | 22 |
| *EMP1* | ENSG00000134531 | ENSRNOG00000008676 | Heart<br>Liver | Coronary artery calcification | 23 |
| *ENG* | ENSG00000106991 | ENSRNOG00000050190 | Heart | Metabolic syndrome | 24 |
| *ETFDH* | ENSG00000171503 | ENSRNOG00000009538 | Heart<br>Liver | Metabolite levels/traits | 10,11 |
| *FADS3* | ENSG00000221968 | ENSRNOG00000020385 | Liver | Metabolite levels<br>HDL/LDL cholesterol<br>Triglycerides<br>Lipid metabolism phenotypes | 13,25–28 |
| *FES* | ENSG00000182511 | ENSRNOG00000011683 | Liver | Diastolic/Systolic Blood pressure<br>Hypertension | 29,30 |
| *GNB4* | ENSG00000114450 | ENSRNOG00000011070 | Heart | Heart rate | 16 |
| *GRB14* | ENSG00000115290 | ENSRNOG00000031396 | Heart | Blood pressure<br>Type 2 diabetes | 31,32 |
| *KIAA1755* | ENSG00000149633 | ENSRNOG00000014424 | Heart | Heart rate | 16 |
| *KLF6* | ENSG00000067082 | ENSRNOG00000016885 | Liver | Coronary artery calcification | 23 |
| *KLF9* | ENSG00000119138 | ENSRNOG00000014215 | Liver | Body mass index | 33 |
| *LAMC2* | ENSG00000058085 | ENSRNOG00000002667 | Heart | Coronary heart disease | 34 |
| *MGMT* | ENSG00000170430 | ENSRNOG00000016038 | Liver | Metabolite levels (X-11787) | 35 |
| *MTCH2* | ENSG00000109919 | ENSRNOG00000008682 | Heart | Body mass index | 36,37 |
| *MYH6* | ENSG00000197616 | ENSRNOG00000025757 | Heart | Heart rate | 16 |
| *NEIL3* | ENSG00000109674 | ENSRNOG00000011688 | Heart | Heart rate variability traits | 38 |
| *NPC1* | ENSG00000141458 | ENSRNOG00000012016 | Liver | Obesity | 39 |
| *PDE11A* | ENSG00000128655 | ENSRNOG00000024457 | Heart | Heart rate | 16 |
| *PLD5* | ENSG00000180287 | ENSRNOG00000003997 | Heart | Coronary artery calcification<br>Obesity-related traits | 18,23 |
| *PLEKHG1* | ENSG00000120278 | ENSRNOG00000016011 | Heart | Blood pressure<br>Obesity-related traits | 18,40 |
| *PLEKHO2* | ENSG00000241839 | ENSRNOG00000029242 | Heart<br>Liver | Coronary heart disease | 34 |
| *PRF1* | ENSG00000180644 | ENSRNOG00000000562 | Heart | Obesity | 41 |
| *PRKCE* | ENSG00000171132 | ENSRNOG00000015603 | Liver | Metabolite levels (X-11787) | 35 |
| *PTPRD* | ENSG00000153707 | ENSRNOG00000005711 | Liver | Type 2 diabetes<br>Obesity-related traits | 18,42,43 |
| *RBM43* | ENSG00000184898 | ENSRNOG00000004673 | Heart | Type 2 diabetes | 44 |
| *SLC1A4* | ENSG00000115902 | ENSRNOG00000005248 | Heart | Metabolite levels | 14,28 |
| *SOX17* | ENSG00000164736 | ENSRNOG00000027357 | Liver | LDL cholesterol | 13 |
| *TNFAIP3* | ENSG00000118503 | ENSRNOG00000049517 | Heart | Cardiac Troponin-T levels | 45 |
| *WDR12* | ENSG00000138442 | ENSRNOG00000017340 | Heart | Myocardial infarction<br>Coronary heart/artery disease | 17,19,46 |

**Supplementary Table 7.** List of primers used for qRT-PCR. F, forward; R, reverse. *Myh6* and *Myh7* share the same forward primer, but the different reverse oligos ensure the amplification of specific products.

| **Gene Symbol** | **F (5‘ – 3‘)** | **R (5‘ – 3‘)** |
|---|---|---|
| *Ctps1* | GTTCCTTGATATCCGCCTCAC | CATCACCCACTCTTGAATTGC |
| *Fads1* | CCCACCAAGAATAAGGCGCT | TTTCATGAGGCCCATTCGCT |
| *Fes* | GCCAGCAAAGACAAGGATCG | AGTACGTAGCGGTTGTGGTG |
| *Gja1* | AGGTCTGAGAGCCTGAACTCT | CATGTCTGGGCACCTCTCTT |
| *Maoa* | AATGGGTAGATGTTGGTGGAG | CCACGGAATGGGTAAGTTTTC |
| *Mrpl48* | ATGAGCGGAACCCTGGGAAAG | CCACCTGCAGAATAAATGGGAT |
| *Myh6* | AGAGGAGAGGGCGGACATTG | AACAGCGAGGCTCTTTCTGC |
| *Myh7* | AGAGGAGAGGGCGGACATTG | GGCATCCTTAGGGTTGGGTAG |
| *Polr2a* | CACTCAAGCTGACGGATTACAGA | GAGCATGGACGCCAAAGC |
| *Ppia* | GCAGACAAAGTTCCAAAGACAG | CCATTATGGCGTGTGAAGTC |
| *Tbp* | TTCGTGCCAGAAATGCTGAA | TTCGTGGCTCTCTTATTCTCATGA |

## References


1. Love, M. I., Huber, W. & Anders, S. Moderated estimation of fold change and dispersion for RNA-seq data with DESeq2. *Genome Biol.* **15,** 550 (2014).
2. Low, T. *et al.* Quantitative and Qualitative Proteome Characteristics Extracted from In-Depth Integrated Genomics and Proteomics Analysis. *Cell Rep* **5,** (2013).
3. Ingolia, N., Ghaemmaghami, S., Newman, J. & Weissman, J. Genome-Wide Analysis in Vivo of Translation with Nucleotide Resolution Using Ribosome Profiling. *Science* **324,** 218–223 (2009).
4. Rintisch, C. *et al.* Natural variation of histone modification and its impact on gene expression in the rat genome. *Genome Res.* **24,** 942–953 (2014).
5. Ray, D. *et al.* A compendium of RNA-binding motifs for decoding gene regulation. *Nature* **499,** 172–177 (2013).
6. Kanehisa, M. & Goto, S. KEGG: kyoto encyclopedia of genes and genomes. *Nucleic acids research* **28,** 27–30 (2000).
7. Duncan, D., Prodduturi, N. & Zhang, B. WebGestalt2: an updated and expanded version of the Web-based Gene Set Analysis Toolkit. *BMC Bioinformatics* **11,** P10 (2010).
8. Kathiresan, S. *et al.* Six new loci associated with blood low-density lipoprotein cholesterol, high-density lipoprotein cholesterol or triglycerides in humans. *Nat. Genet.* **40,** 189–97 (2008).
9. Hong, M.-G. G. *et al.* A genome-wide assessment of variability in human serum metabolism. *Hum. Mutat.* **34,** 515–24 (2013).
10. Illig, T. *et al.* A genome-wide perspective of genetic variation in human metabolism. *Nat. Genet.* **42,** 137–41 (2010).
11. Suhre, K. *et al.* Human metabolic individuality in biomedical and pharmaceutical research. *Nature* **477,** 54–60 (2011).
12. Newton-Cheh, C. *et al.* Common variants at ten loci influence QT interval duration in the QTGEN Study. *Nat. Genet.* **41,** 399–406 (2009).
13. Willer, C. J. *et al.* Discovery and refinement of loci associated with lipid levels. *Nat. Genet.* **45,** 1274–83 (2013).
14. Inouye, M. *et al.* Novel Loci for metabolic networks and multi-tissue expression studies reveal genes for atherosclerosis. *PLoS Genet.* **8,** e1002907 (2012).
15. He, J. *et al.* Genome-wide association study identifies 8 novel loci associated with blood pressure responses to interventions in Han Chinese. *Circ Cardiovasc Genet* **6,** 598–607 (2013).
16. Hoed, M. den *et al.* Identification of heart rate-associated loci and their effects on cardiac conduction and rhythm disorders. *Nat. Genet.* **45,** 621–31 (2013).
17. Schunkert, H. *et al.* Large-scale association analysis identifies 13 new susceptibility loci for coronary artery disease. *Nat. Genet.* **43,** 333–8 (2011).
18. Comuzzie, A. G. *et al.* Novel genetic loci identified for the pathophysiology of childhood obesity in the Hispanic population. *PLoS ONE* **7,** e51954 (2012).
19. Dichgans, M. *et al.* Shared genetic susceptibility to ischemic stroke and coronary artery disease: a genome-wide analysis of common variants. *Stroke* **45,** 24–36 (2014).
20. O'Donnell, C. J. *et al.* Genome-wide association study for coronary artery calcification with follow-up in myocardial infarction. *Circulation* **124,** 2855–64 (2011).
21. Yuan, X. *et al.* Population-based genome-wide association studies reveal six loci influencing plasma levels of liver enzymes. *Am. J. Hum. Genet.* **83,** 520–8 (2008).
22. Marroni, F. *et al.* A genome-wide association scan of RR and QT interval duration in 3 European genetically isolated populations: the EUROSPAN project. *Circ Cardiovasc Genet* **2,** 322–8 (2009).

23. Wojczynski, M. *et al.* Genetics of coronary artery calcification among African Americans, a meta-analysis. *BMC Med. Genet.* **14,** 75 (2013).
24. Zabaneh, D. & Balding, D. A Genome-Wide Association Study of the Metabolic Syndrome in Indian Asian Men. *PLoS ONE* **5,** (2010).
25. Xie, W. *et al.* Genetic Variants Associated With Glycine Metabolism and Their Role in Insulin Sensitivity and Type 2 Diabetes. *Diabetes* **62,** 2141–2150 (2013).
26. Kathiresan, S. *et al.* Common variants at 30 loci contribute to polygenic dyslipidemia. *Nat. Genet.* **41,** 56–65 (2008).
27. Aulchenko, Y. *et al.* Loci influencing lipid levels and coronary heart disease risk in 16 European population cohorts. *Nat. Genet.* **41,** 47–55 (2008).
28. Kettunen, J. *et al.* Genome-wide association study identifies multiple loci influencing human serum metabolite levels. *Nat. Genet.* **44,** 269–76 (2012).
29. Wain, L. *et al.* Genome-wide association study identifies six new loci influencing pulse pressure and mean arterial pressure. *Nat. Genet.* **43,** 1005–1011 (2011).
30. Ehret, G. B.*et al.* Genetic variants in novel pathways influence blood pressure and cardiovascular disease risk. *Nature* **478,** 103–109 (2011).
31. Kato, N. *et al.* Meta-analysis of genome-wide association studies identifies common variants associated with blood pressure variation in east Asians. *Nat. Genet.* **43,** 531–538 (2011).
32. Kooner, J. *et al.* Genome-wide association study in individuals of South Asian ancestry identifies six new type 2 diabetes susceptibility loci. *Nat. Genet.* **43,** 984–989 (2011).
33. Okada, Y. *et al.* Common variants at CDKAL1 and KLF9 are associated with body mass index in east Asian populations. *Nat. Genet.* **44,** 302–306 (2012).
34. Lettre, G. *et al.* Genome-Wide Association Study of Coronary Heart Disease and Its Risk Factors in 8,090 African Americans: The NHLBI CARe Project. *PLoS Genet.* **7,** (2011).
35. Yu, B. *et al.* Genome□Wide Association Study of a Heart Failure Related Metabolomic Profile Among African Americans in the Atherosclerosis Risk in Communities (ARIC) Study. *Genet. Epidemiol.* **37,** 840–845 (2013).
36. Willer, C. *et al.* Six new loci associated with body mass index highlight a neuronal influence on body weight regulation. *Nat. Genet.* **41,** 25–34 (2008).
37. Speliotes, E. *et al.* Association analyses of 249,796 individuals reveal 18 new loci associated with body mass index. *Nat. Genet.* **42,** 937–948 (2010).
38. Newton-Cheh, C. *et al.* Genome-wide association study of electrocardiographic and heart rate variability traits: the Framingham Heart Study. *BMC Med. Genet.* **8 Suppl 1,** S7 (2007).
39. Meyre, D. *et al.* Genome-wide association study for early-onset and morbid adult obesity identifies three new risk loci in European populations. *Nat. Genet.* **41,** 157–159 (2009).
40. Franceschini, N. *et al.* Genome-wide Association Analysis of Blood-Pressure Traits in African-Ancestry Individuals Reveals Common Associated Genes in African and Non-African Populations. *Am. J. Hum. Genet.* **93,** (2013).
41. Cotsapas, C. *et al.* Common body mass index-associated variants confer risk of extreme obesity. *Hum. Mol. Genet.* **18,** 3502–7 (2009).
42. Below, J. E. *et al.* Genome-wide association and meta-analysis in populations from Starr County, Texas, and Mexico City identify type 2 diabetes susceptibility loci and enrichment for expression quantitative trait loci in top signals. *Diabetologia* **54,** 2047–55 (2011).
43. Tsai, F.-J. J. *et al.* A genome-wide association study identifies susceptibility

variants for type 2 diabetes in Han Chinese. *PLoS Genet.* **6,** e1000847 (2010).
44. Palmer, N. D. *et al.* A genome-wide association search for type 2 diabetes genes in African Americans. *PLoS ONE* **7,** e29202 (2012).
45. Yu, B. *et al.* Association of genome-wide variation with highly sensitive cardiac troponin-T levels in European Americans and Blacks: a meta-analysis from atherosclerosis risk in communities and cardiovascular health studies. *Circ Cardiovasc Genet* **6,** 82–8 (2013).
46. Kathiresan, S. *et al.* Genome-wide association of early-onset myocardial infarction with single nucleotide polymorphisms and copy number variants. *Nat. Genet.* **41,** 334–41 (2009).
